# Supplementary material for: Differential Membrane Binding and Seeding of Distinct α-Synuclein Fibrillar Polymorphs
Source: Biophys J. 2020 Jan 28;118(6):1301–20. doi: 10.1016/j.bpj.2020.01.022 (PMC7091477; doi:10.1016/j.bpj.2020.01.022)
Supplement: Document S1. Figs. S1–S8 [file mmc1.pdf]

Biophysical Journal, Volume *118*

Supplemental Information

Differential Membrane Binding and Seeding of Distinct α-Synuclein Fibrillar Polymorphs

Amulya Nidhi Shrivastava, Luc Bousset, Marianne Renner, Virginie Redeker, Jimmy Savistchenko, Antoine Triller, and Ronald Melki

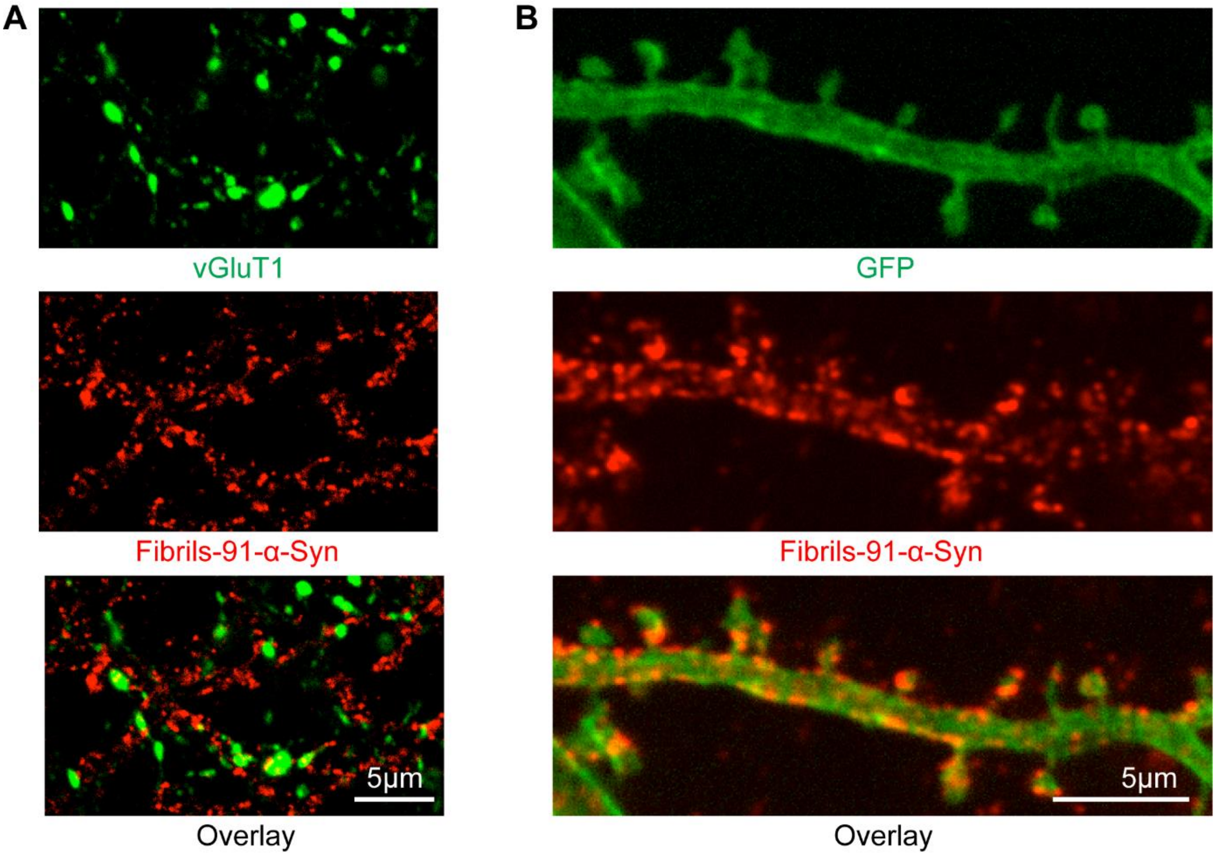

Supplementary Figure 1. Binding of α-Syn in and out of synapses

(**A**) Exposure of neurons to fibrillar α-Syn polymorphs (image for Fibrils-91-ATTO550 is presented, red) followed by immunolabeling of excitatory pre-synaptic boutons using vGluT1 (green) antibody. Binding and clustering of α-Syn is observed both in and out of synaptic terminals.

(**B**) Binding of α-Syn polymorphs (image for Fibrils-91-ATTO550 is presented, red) on neuronal dendrite (labeled with GFP, green). Binding and clustering of α-Syn is observed both in dendritic spines and shaft.

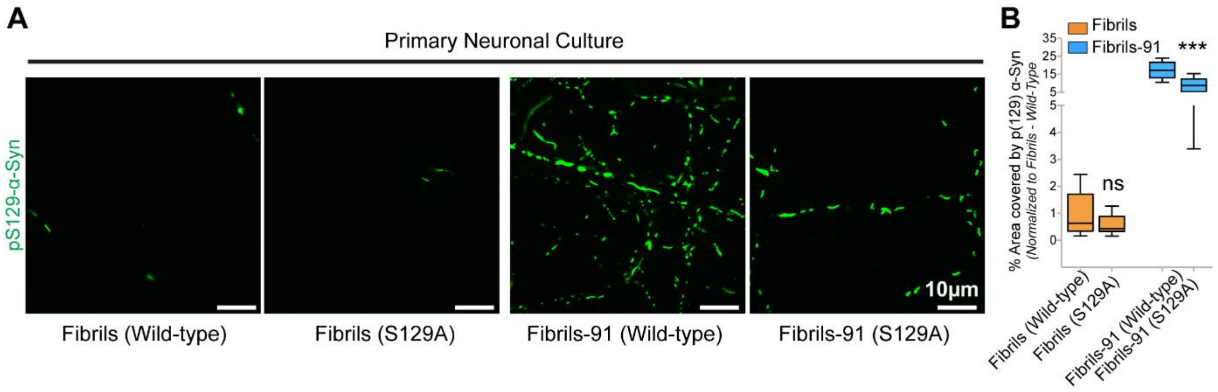

Supplementary Figure 2. Endogenous α-Syn is phosphorylated upon exposure in primary neuronal cultures to exogenous fibrillar polymorphs

(**A-B**) Primary mature hippocampal neurons cultures were exposed to wild-type or S129A fibrillar polymorphs (Fibrils and Fibrils-91) (250nM, 15 min in fresh culture medium) at DIV 14. After extensive washing, cells were transferred back to the original culture medium. Neurons were fixed at DIV 21 and immunolabeled for pS129-α-Syn (**A**). Similar pS129-α-Syn signal was observed after exposure to wild-type or S129A fibrillar α-Syn polymorphs (**A**). Quantification of the area occupied by aggregated pS129-α-Syn using the 81A antibody following exposure to the fibrillar α-Syn polymorphs.

Box-plot shows median, inter-quartile range and 10-90% distribution. Mann-Whitney test is performed to compare the difference; number of images (n) from 2 experiments (left to right): 16, 22, 30, 30. ***p<0.001, ns= not significant.

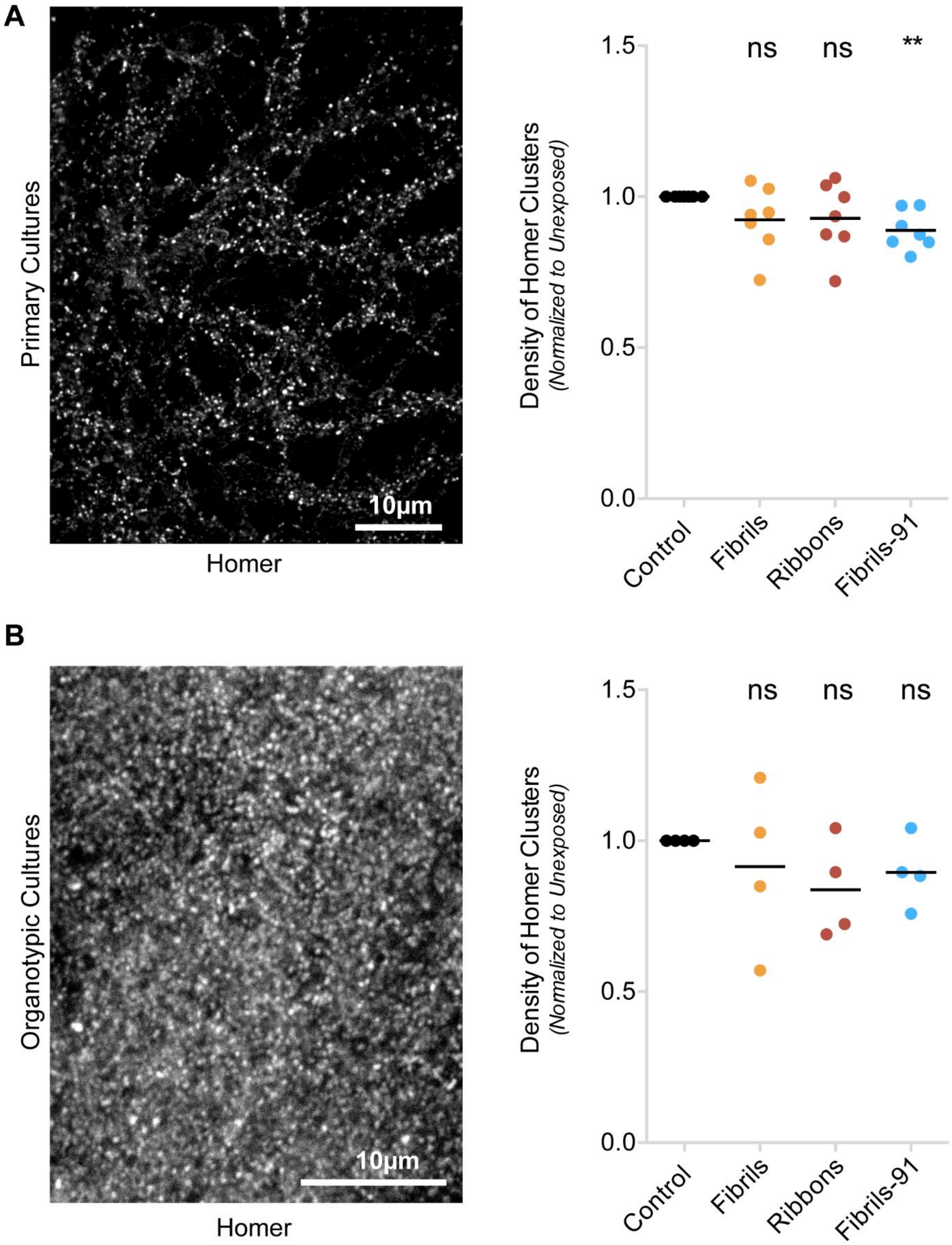

Supplementary Figure 3. No alteration in synapse density in α-Syn seeded neurons

(**A-D**) Immunodetection of synapses using Homer antibody in primary neurons (**A**) or organotypic slices (**B**) in control or α-Syn polymorphs seeded neurons. No or subtle alteration in synaptic density was observed (**B, D**). Dot plot shows average value from 7-experiments in cultures and 4-experiments in slices; Mann-Whitney test, **$p<0.01$, ns= not significant.

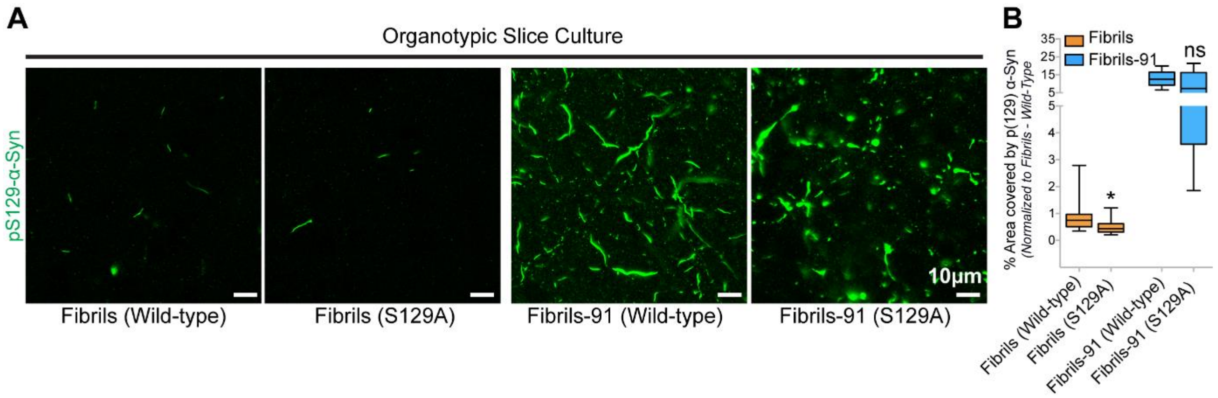

Supplementary Figure 4. Endogenous α-Syn is phosphorylated upon exposure of organotypic slice cultures to exogenous fibrillar polymorphs

(**A-B**) Organotypic slice cultures were exposed to wild-type or S129A fibrillar polymorphs (Fibrils and Fibrils-91 polymorphs) (1.5µM, 15 min in fresh culture medium) at day 0. After extensive washing, slices were transferred to a new culture medium. Slices were fixed on day 14 and immunolabeled for pS129-α-Syn (**A**). Similar pS129-α-Syn signal was observed after exposure to wild-type or S129A fibrillar α-Syn polymorphs (**A**). Quantification of the area occupied by aggregated pS129-α-Syn using the 81A antibody following exposure to the fibrillar α-Syn polymorphs.

Box-plot shows median, inter-quartile range and 10-90% distribution. Mann-Whitney test is performed to compare the difference; number of images (n) from 2 experiments (left to right): 22, 21, 22, 23. *p<0.05, ns= not significant.

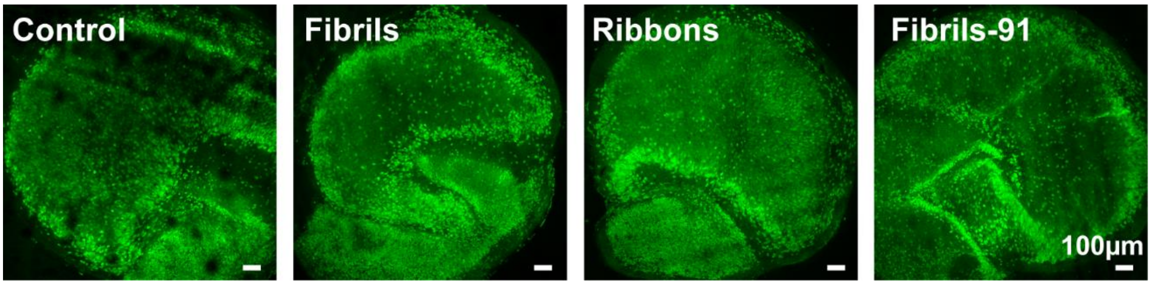

Supplementary Figure 5. Morphology of organotypic slices in seeded neurons

Low magnification images of brain slices immunostained for neuronal marker, NeuN (green) prior (control) or after exposure to α-Syn polymorphs on Day 28 in culture after addition of the fibrillar polymorphs on Day 14. No alteration in hippocampal morphology is detected.

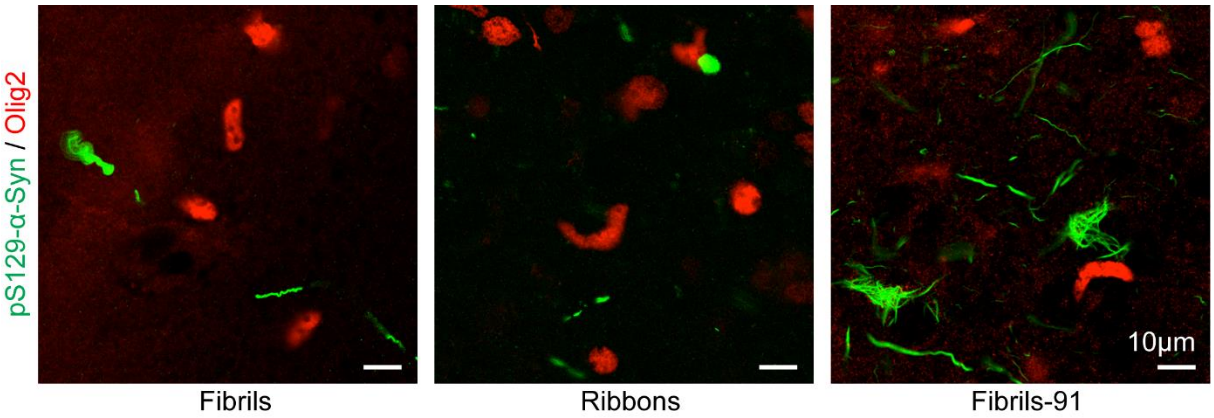

Supplementary Figure 6. Detection of pS129-α-Syn aggregates in oligodendrocytes

Organotypic slice cultures were exposed to the fibrillar polymorphs Fibrils, Ribbons and Fibrils-91(1.5µM) at day 0. Slices were fixed on day 14 and immunolabeled for pS129-α-Syn (81A antibody) and oligodendrocyte marker (Olig 2 antibody). No pS129-α-Syn deposits in Olig2 positive cells for slices exposed to Fibrils/Fibrils-91 polymorphs was observed. Occasional pS129-α-Syn reactivity within Olig2-positive oligodendrocytes was observed for Ribbons (middle).

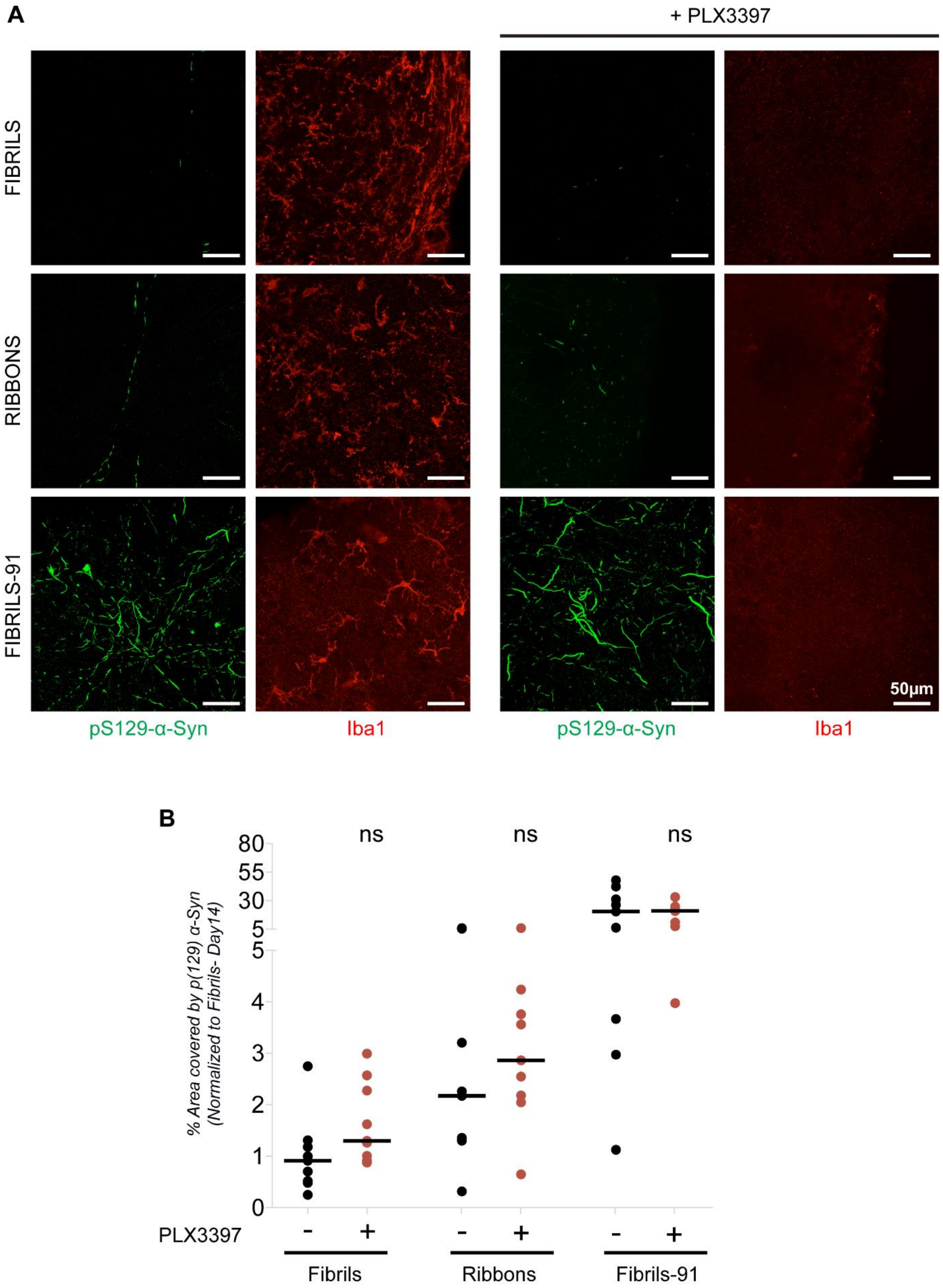
A
+ PLX3397
FIBRILS
RIBBONS
FIBRILS-91
50µm
pS129-α-Syn
Iba1
pS129-α-Syn
Iba1
B
ns
ns
ns
% Area covered by p(129) α-Syn
(Normalized to Fibrils- Day14)
80
55
30
5
5
4
3
2
1
0
PLX3397
-
+
-
+
-
+
Fibrils
Ribbons
Fibrils-91

Supplementary Figure 7. Unaltered seeding in organotypic slices following microglia depletion

(**A-B**) Organotypic slice cultures were prepared (day -14) and exposed to α-Syn fibrillar polymorphs (1.5μM, 15 min in fresh culture medium) at day 0. After extensive washing, the slices were transferred to a new culture medium. Slices were fixed on day 14 and immunolabeled for pS129-α-Syn and Iba1. Complete microglia depletion (**A**) was achieved using PLX3397 treatment as described in Materials and Methods. Notably fibrillar polymorphs seeded the aggregation of endogenous α-Syn both in absence and presence of microglia (**A, B**). Dot-plot shows individual organotypic slice. Mann-Whitney test is performed to compare the difference; number of slices (n=10) from 3 experiments. *$p<0.05$, ns= not significant.

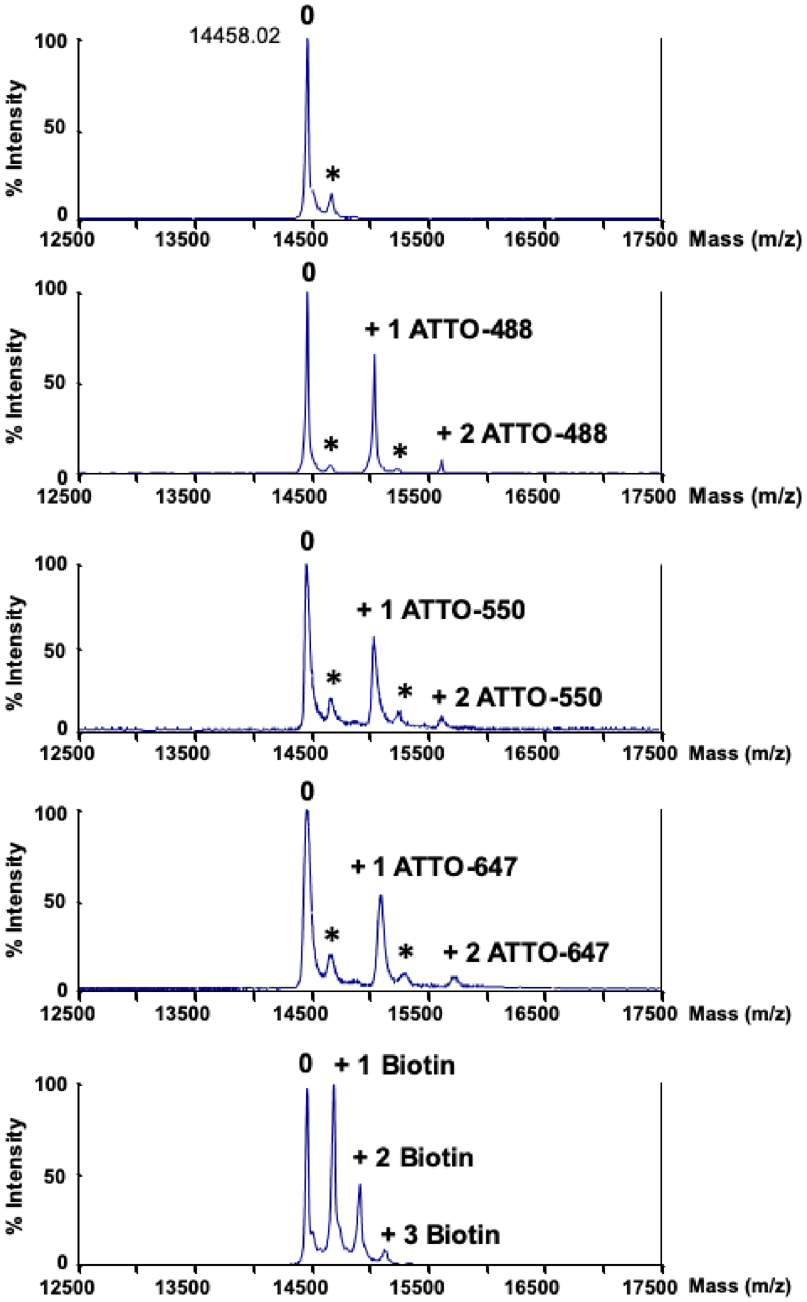
0
14458.02
100
50
0
% Intensity
*
12500
13500
14500
15500
16500
17500
Mass (m/z)
0
+ 1 ATTO-488
+ 2 ATTO-488
0
+ 1 ATTO-550
+ 2 ATTO-550
0
+ 1 ATTO-647
+ 2 ATTO-647
0
+ 1 Biotin
+ 2 Biotin
+ 3 Biotin

Supplementary Figure 8. Quality control analysis of labeled α-Syn Fibrils . MALDI-TOF mass spectra, from top to bottom of (A) unlabelled, (B) ATTO480, (C) ATTO550, (D) ATTO647 and (E) biotin-labeled wild-type α-Syn Fibrils are shown. The spectra show that α-Syn is labelled on average by ≤ one ATTO480, 550, 647 or biotin molecule. Stars depict sinapinic acid matrix adducts.

For mass spectrometry analysis, the samples were de-salted (with 5% acetonitrile, 0.1% Trifluoroacetic acid (TFA)) and eluted from a C18 reversed-phase Zip-Tip (Millipore, Billerica, MA, USA) in 50% acetonitrile, 0.1% TFA. The polypeptides were mixed in a ratio of 1:5 to 1:20 (v⁄v) with sinapinic acid (10 mg/mL) in 50% acetonitrile and 0.1% TFA) and spotted (0.5 µL) on a stainless steel MALDI target (Opti-TOF; Applied BioSystems). MALDI-TOF-TOF MS spectra were acquired with a MALDI-TOF⁄TOF 5800 mass spectrometer (Applied Biosystems) using linear mode acquisition. Acquisition and data analysis were performed using the Data Explorer software from Applied Biosystems.
