# Supplementary material for: Differential Membrane Binding and Seeding of Distinct α-Synuclein Fibrillar Polymorphs
Source: Biophys J. 2020 Jan 28;118(6):1301–20. doi: 10.1016/j.bpj.2020.01.022 (PMC7091477; doi:10.1016/j.bpj.2020.01.022)
Supplement: Document S2. Article plus Supporting Material [file mmc2.pdf]

Biophysical Journal
Article

Differential Membrane Binding and Seeding of Distinct α-Synuclein Fibrillar Polymorphs

Amulya Nidhi Shrivastava,[1,*] Luc Bousset,[1] Marianne Renner,[2] Virginie Redeker,[1] Jimmy Savistchenko,[1] Antoine Triller,[3,*] and Ronald Melki[1,*]

[1]CEA, Institut François Jacob (MIRcen) and CNRS, Laboratory of Neurodegenerative Diseases, Fontenay-aux-Roses, France; [2]INSERM, UMR- S 839 Institut du Fer à Moulin, Sorbonne Université, Paris, France; and [3]École Normale Supérieure, Institut de Biologie de l'ENS, INSERM, CNRS, PSL, Research University, Paris, France

ABSTRACT The aggregation of the protein α-synuclein (α-Syn) leads to different synucleinopathies. We recently showed that structurally distinct fibrillar α-Syn polymorphs trigger either Parkinson's disease or multiple system atrophy hallmarks in vivo. Here, we establish a structural-molecular basis for these observations. We show that distinct fibrillar α-Syn polymorphs bind to and cluster differentially at the plasma membrane in both primary neuronal cultures and organotypic hippocampal slice cultures from wild-type mice. We demonstrate a polymorph-dependent and concentration-dependent seeding. We show a polymorph-dependent differential synaptic redistribution of α3-Na^{+}/K^{+}-ATPase, GluA2 subunit containing α-amino-3-hydroxy-5-methyl-4-isoxazolepropionic acid receptors, and GluN2B-subunit containing N-methyl-D-aspartate receptors, but not GluA1 subunit containing α-amino-3-hydroxy-5-methyl-4-isoxazolepropionic acid and metabotropic glutamate receptor 5 receptors. We also demonstrate polymorph-dependent alteration in neuronal network activity upon seeded aggregation of α-Syn. Our findings bring new, to our knowledge, insight into how distinct α-Syn polymorphs differentially bind to and seed monomeric α-Syn aggregation within neurons, thus affecting neuronal homeostasis through the redistribution of synaptic proteins.

SIGNIFICANCE Different synucleinopathies (Parkinson's disease, multiple system atrophy, dementia with Lewy bodies) are the consequence of the aggregation of α-synuclein (α-Syn) into high-molecular-weight assemblies that possess distinct intrinsic structures (polymorphs). This study shows that distinct fibrillar α-Syn polymorphs' initial binding and clustering to the plasma membrane are tightly linked to subsequent pS129-α-Syn aggregate accumulation. Distinct fibrillar α-Syn polymorphs differentially redistribute synaptic proteins and differentially alter neuronal network activity. This study suggests that distinct α-Syn fibrillar polymorphs' differential interaction with cellular components leads to distinct synucleinopathies.

INTRODUCTION

Synucleinopathies are a class of neurodegenerative diseases that have in common the aggregation of the protein α-synuclein (α-Syn). They comprise Parkinson's disease (PD) without or with dementia, dementia with Lewy bodies, multiple system atrophy (MSA), and Gaucher disease (1,2). It has been proposed that different synucleinopathies are the consequence of the aggregation of α-Syn into high-molecular-weight assemblies that possess distinct intrinsic structures (2,3). Indeed, α-Syn's chameleon property yields multiple conformations, allowing the formation of fibrillar assemblies with distinct structures and surfaces that dictate their growth and clearance propensities. Experimental evidence for polymorph-pathology interdependence came from recent works (4–7) in which different α-Syn fibrillar polymorph injections in rodent brains yielded phenotypes characteristic of PD and MSA.

The distinct intrinsic architectures different α-Syn polymorphs possess are due to the different amino acid stretches involved in their amyloid cores (5). Given that different amino acid stretches are involved in distinct α-Syn polymorph amyloid cores (5,8), those they expose at their surfaces also differ (9). The tip and the side surfaces of

Submitted September 25, 2019, and accepted for publication January 15, 2020.

*Correspondence: amulya.nidhi@gmail.com or triller@biologie.ens.fr or ronald.melki@cnrs.fr

Amulya Nidhi Shrivastava's present address is UCB Biopharma SRL, 1420 Braine Braine l'Alleud, Belgium.

Editor: Joseph Falke.

https://doi.org/10.1016/j.bpj.2020.01.022

© 2020 Biophysical Society.

This is an open access article under the CC BY license (http://creativecommons.org/licenses/by/4.0/).

pathogenic α-Syn fibrillar assemblies possess have to be considered separately. The growing ends define the rate at which they elongate by recruitment of monomeric α-Syn in conformations that can establish highly complementary interactions. The amino acid stretches exposed on the sides of distinct α-Syn polymorphs define what membranous components, in particular plasma membrane proteins, they can interact with. Recent studies reported the interaction of exogenous fibrillar α-Syn with extracellularly exposed membrane proteins (10,11). The presence of those protein partners and their abundance on the neuronal plasma membrane define the tropism of distinct α-Syn polymorphs toward cell populations within the central nervous system.

After binding and uptake of fibrillar α-Syn, seeding occurs (12). This is accompanied in cell cultures and in vivo by post-translational modifications ranging from proteolytic cleavages to phosphorylation, ubiquitination, etc. of newly aggregated endogenous α-Syn. Efficient seeding of neuronal α-Syn by exogenous fibrils has been demonstrated (13). Organotypic slice cultures represent a powerful alternative to primary neuronal cultures because they allow assessing seeding and propagation of infectious proteins in a context in which neuronal circuits are maintained partially intact. Organotypic slices have been indeed widely used to study prion pathology (14–16). Here, we show that five distinct fibrillar α-Syn polymorphs (fibrils, ribbons, fibrils-91, fibrils-65, and fibrils-110) bind to and cluster differentially at the plasma membrane in both primary neuronal cultures and organotypic hippocampal slice cultures from wild-type (WT) mice. We demonstrate a polymorph-dependent and concentration-dependent seeding. We prove that a fibrillar polymorph's initial binding and clustering to the plasma membrane is tightly linked to subsequent pS129-α-Syn aggregate accumulation. We also report a fibrillar-polymorph-dependent differential synaptic redistribution of the α3-subunit of sodium/potassium-ATPase (α3-NKA), GluA2 subunit containing α-amino-3-hydroxy-5-methyl-4-isoxazolepropionic acid (GluA2-AMPA) receptors, and GluN2B-subunit containing N-methyl-D-aspartate (GluN2B-NMDA) receptors. We also demonstrate fibrillar-polymorph-dependent alteration in neuronal network activity upon seeded aggregation of α-Syn. Altogether, our findings suggest that distinct α-Syn fibrillar polymorphs differentially affect neuronal homeostasis.

MATERIALS AND METHODS

Generation, labeling, and characterization of fibrillar α-Syn polymorphs

The expression and purification of human WT α-Syn was performed as previously described (17). A variant human α-Syn in which serine 129 residue was changed to alanine (S129A α-Syn) was generated by site-directed mutagenesis. This variant cannot be phosphorylated in neurons on S129, the main phosphorylation site for α-Syn. S129A α-Syn was purified exactly like WT α-Syn. WT α-Syn or S129A α-Syn was incubated in buffer A to obtain the fibrillar polymorph "fibrils" (50 mM Tris-HCl (pH 7.5), 150 mM KCl), in buffer B for "ribbons" (5 mM Tris-HCl (pH 7.5)), in buffer C for "fibrils-65" (20 mM 2-(N-morpholino)ethanesulfonic acid hydrate (pH 6.5), 150 mM NaCl), and in buffer D for "fibrils-91" (20 mM KPO4 (pH 9.1)) at 37°C under continuous shaking in an Eppendorf Thermomixer (Hamburg, Germany) set at 600 rotations per minute (rpm) for 4–7 days (5,18). A truncated human α-Syn spanning residues 1–110 was generated by introducing two stop codons after residue 110 by site-directed mutagenesis. This variant was purified exactly like full-length α-Syn and was assembled into the fibrillar structure "fibrils-110" in buffer A (50 mM Tris-HCl (pH 7.5), 150 mM KCl). The fibrillar α-Syn polymorphs were centrifuged twice at 15,000 × *g* for 10 min and resuspended twice in phosphate-buffered saline (PBS) at 1,446 g/L. All preformed assemblies were labeled with ATTO-488 NHS-ester, ATTO-550 NHS-ester, or ATTO-647N NHS-ester (#AD 488-3, AD 550-35, and AD 647N-35, respectively; Atto-Tec, Siegen, Germany) fluorophore following the manufacturer's instructions and/or biotin using EZ-link Sulfo-NHS-Biotin (sulfosuccinimidobiotin; Perbio Science, Cramlington, UK) using a protein/dye tag ratio of 1:2. The labeling reactions were arrested by addition of 1 mM Tris (pH 7.5). The unreacted fluorophore was removed by a final cycle of two centrifugations at 15,000 × *g* for 10 min and resuspensions of the pellets in PBS. Mass spectrometry was used to quantify the number of incorporated ATTO or biotin molecules per α-Syn monomer within the fibrillar assemblies as previously described (10,19). This labeling protocol typically yields ≤1 ATTO or biotin molecule incorporated per α-Syn monomer on average (Fig. S8). The quality control of human recombinant monomeric WT or S129A α-Syn and the fibrillar polymorphs they generate and that of α-Syn 1–110 was carried out as previously described (5,18). The fibrillar polymorphs were fragmented by sonication for 20 min in 2-mL Eppendorf tubes in a Vial Tweeter powered by an ultrasonic processor UIS250v (250 W, 2.4 kHz; Hielscher Ultrasonic, Teltow, Germany) to generate fibrillar particles with an average size of 42–52 nm that are suitable for endocytosis.

For transmission electron microscopy, the assemblies were adsorbed on 200 mesh carbon-coated electron microscopy grids and imaged after negative staining with 1% uranyl acetate before and after fragmentation using a JEOL 1400 electron microscope (JEOL, Tokyo, Japan). For fibrillar polymorph fingerprint analysis, we used degradation by proteinase K. Aliquots of fibrillar assemblies were removed before or after addition of proteinase K (3.8 μg mL^{-1}), denatured in boiling Laemmli buffer for 5 min at 90°C, subjected to sodium dodecyl sulfate-polyacrylamide gel electrophoresis (SDS-PAGE) on 12% polyacrylamide gels, and stained by Coomassie coloration.

Organotypic slice culture and protocol for seeding with fibrillar α-Syn polymorphs

Slice were cultured in MEM (minimal essential medium, Thermo Fisher Scientific, Waltham, MA) medium supplemented with 20% heat-inactivated horse serum (Eurobio, Les Ulis, France), 2 mM Glutamax 100 (Thermo Fisher Scientific), 1 mM $CaCl_2$, 2 mM $MgSO_4$, 2 mM $MgCl_2$, 11 mM d-Glucose, 5 mM $NaHCO_3$ (Thermo Fisher Scientific), and 20 mM Hepes (Thermo Fisher Scientific) (20). Before dissection, six-well dishes were prepared with 900 μL of culture media with a millicell insert (30-mm biopore polytetrafluoroethylene membrane, type CM, 0.4 μm; Millipore, Burlington, MA). Hippocampi were dissected from P3 to P5 C57BL/6 mice and kept on ice in PBS (1×)-glucose (1×) solution. Hippocampi were sliced (400 μm) using a McIllwain tissue chopper (Mickle Laboratory, Gainesville, FL) and separated in the prewarmed culture medium. Four to six slices were plated on millicell inserts in each six-well dishes. Slices were maintained for 28 days, with medium changed twice a week. Exposure to α-Syn fibrillar polymorphs was performed on day 14. Fibrillar α-Syn polymorphs were diluted in fresh culture medium and applied on top of the slices for 15 min, followed by three washes with culture medium. The millicells were then transferred to new

six-well dishes containing fresh medium. Microglial cells were depleted using colony-stimulating factor 1 receptor inhibitor, PLX3397. The inhibitor was supplemented in the culture medium (10 μM) starting from day 7 until the end of the experiment, i.e., day 28. Notably, by day 14, when fibrillar α-Syn exposure was performed, microglial cells were completely depleted.

Exposure concentrations

The fibrillar polymorph concentration is expressed throughout the work as monomer-equivalent concentration. The amount of aggregated α-Syn within all fibrillar polymorphs was determined by ultracentrifugation and measurement of the amount of monomeric α-Syn in the supernatant. The fibrillar assemblies were fragmented to the similar size (Fig. 1, *A* and *B*). In primary neurons, short-term experiments (binding and dynamics) were performed at 50 nM concentration. Seeding experiments were performed at 250 nM (15 min exposure, followed by wash) because no seeding was detected for the fibril polymorph at 50 nM concentration. For organotypic slices, seeding experiments were performed at 750 and 1500 nM (15 min exposure, followed by wash). Control conditions refer to 1× PBS buffer exposure.

Primary neuronal culture and protocol for seeding with fibrillar α-Syn polymorphs

Primary neuronal culture was performed as described previously (10,21,22). Freshly dissociated (trypsin) hippocampi were plated (10^5 cells/well in a 12-well dish containing an 18-mm coverslip) in neuronal attachment media consisting of 10% horse serum (Eurobio), 1 mM sodium pyruvate (Thermo Fisher Scientific), 2 mM Glutamax-100X (Thermo Fisher Scientific), and penicillin/streptomycin (Thermo Fisher Scientific) in MEM (Thermo Fisher Scientific) for 3 h. The attachment medium was replaced, and cells were maintained in serum-free neurobasal medium (Thermo Fisher Scientific) supplemented with B27 (Gibco, Gaithersburg, MD) and 2 mM Glutamax-100X. Exposure to fibrillar α-Syn polymorphs was performed at days in vitro 14 (DIV 14). Fibrillar α-Syn polymorphs were diluted in fresh neurobasal medium. The "cell-conditioned neurobasal medium" was replaced with fibrillar α-Syn containing neurobasal medium for 15 min, the former kept aside at 37°C. After 15 min exposure, fibrillar-α-Syn-containing medium was removed, and the well was washed thrice. Lastly, the cells were replenished with "cell-conditioned neurobasal medium" and transferred back to the incubator until DIV 21.

Immunolabeling, antibodies used, and quantifications

Immunolabeling was performed as per standard protocol. The antibodies are listed in the table below. For organotypic slices, staining was performed on free-floating sections. For this, slices were detached from millicells, followed by blocking (0.25% gelatin and 0.2% Triton X-100 in 1× PBS) for 45 min. Slices were then incubated overnight with the appropriate primary antibodies diluted in 0.125% gelatin and 0.2% Triton X-100 in 1× PBS. After three washes (20 min each), slices were incubated with secondary antibodies (1:1000, 3 h). After washing for 2–3 h, sections were mounted onto glass slides using Vectashield (Vector Labs, Burlingame, CA). Images were acquired using a Leica confocal TCS SP8 microscope (Leica, Wetzlar, Germany) and processed using ImageJ, Metamorph (Molecular Devices, San Jose, CA), and MATLAB (The MathWorks, Natick, MA).

For primary neurons, blocking (3% bovine serum albumin (BSA) in 1× PBS) was performed for 30 min postpermeabilization (0.2% Triton X-100 in 3% BSA in 1× PBS, 10 min). No permeabilization step was required for methanol fixation. Cells were then incubated in appropriate primary antibodies diluted in 3% BSA in 1× PBS for 1 h. After three washes (10 min each), slices were incubated with secondary antibodies (1:400, 45 min). After three washes (20 min), sections were mounted onto glass slides using Vectashield (Vector Labs). Images were acquired using a Leica inverted spinning disk microscope (DM5000B; Coolsnap HQ2 camera, BioImaging Solutions, San Diego, CA; Cobolt Lasers, Solna, Sweden) (Table 1).

Thresholding is based on wavelet-based segmentation as previously described (10,21–23). Individual structures were identified (i.e., p129-α-Syn aggregates, receptors, and synaptic clusters) to generate background-free masks. The fluorescence intensity of the original images on top of these masks were then computed. For p129-α-Syn, the sum of "intensity of all structures" was calculated. For the two-color analysis, "colocalization" is defined when there was an overlap between the thresholded clusters of two images. "Intensity of cluster" is defined as total fluorescence intensity per cluster. For each image, the values of all clusters were averaged within the field.

Endocytosis assay

To distinguish between cell-surface and internalized spots, α-Syn fibrils were tagged with biotin + ATTO488 dyes. Neuronal conditioned medium was removed (stored at 37°C) and replaced with fresh neurobasal culture medium 15 min before exposure. Live neurons were exposed with fibrillar polymorphs for 1 h in an incubator, followed by three washes with pre-warmed culture medium. The cells were allowed to recover (4 or 8 h) or not (0 h) in neuronal conditioned medium. At the end of each time point, cells were fixed using cold 4% paraformaldehyde (PFA) on ice to prevent membrane rupture due to fixation. Cell-surface biotin + ATTO488 were revealed using streptavidin555 (1:1000, 10 min) followed by extensive washes. Spots positive for biotin-streptavidin555 and ATTO-488 are localized at the cell surface, whereas ATTO4-88-only spots represent endocytosed spots. Quantification was performed as described in the previous paragraph.

Sarkosyl extraction

Neurons were plated on a 10-cm dish and exposed to α-Syn polymorphs (250 nM) on DIV 14 and harvested on DIV 21. Cells were washed one time in ice-cold PBS (1×) and then scraped in 1 mL 1× PBS. The cells were pelleted, and 500 μL extraction buffer was added. Extraction buffer was composed of 20 mM Tris-HCl (pH 7.5), 0.8 M NaCl, 1 mM EGTA, 10% (w/v) sucrose, and 1% sarkosyl supplemented with protease (Roche, Basel, Switzerland) and phosphatase (Sigma-Aldrich, St. Louis, MO) inhibitor cocktails as described recently (24). The cell suspension was incubated at 37°C, gently shaken at 300 rpm for 30 min, and then centrifuged at 1000 rpm for 20 min. The supernatant was collected and probed by Western blotting. 12% gels without stacking layer were used. The following antibodies were used: p129-α-Syn (81A, MABN826, 1:1000; Millipore); mouse-specific α-Syn (D37A6, 4179S, 1:1000; Cell Signaling Technology, Danver, MA), and tubulin (DM1A, Ab7291, 1:2500; Abcam, Cambridge, UK).

Stochastic optical reconstruction microscopy imaging and quantifications

Stochastic optical reconstruction microscopy (STORM) imaging was performed on exogenous fibrillar α-Syn polymorphs labeled with ATTO-647N or on endogenous mouse p129-α-Syn inclusions labeled with mouse monoclonal antibody (primary antibody: 81A, secondary antibody: Alexa647). Imaging was performed under reducing condition with buffer composed of PBS (pH 7.4), glucose (10%), β-mercaptoethylamine (10 mM), glucose oxidase (0.5 mg/mL), and catalase (40 mg/mL) and deoxygenized with nitrogen (10). A total of 20,000 (ATTO-647N) or 40,000

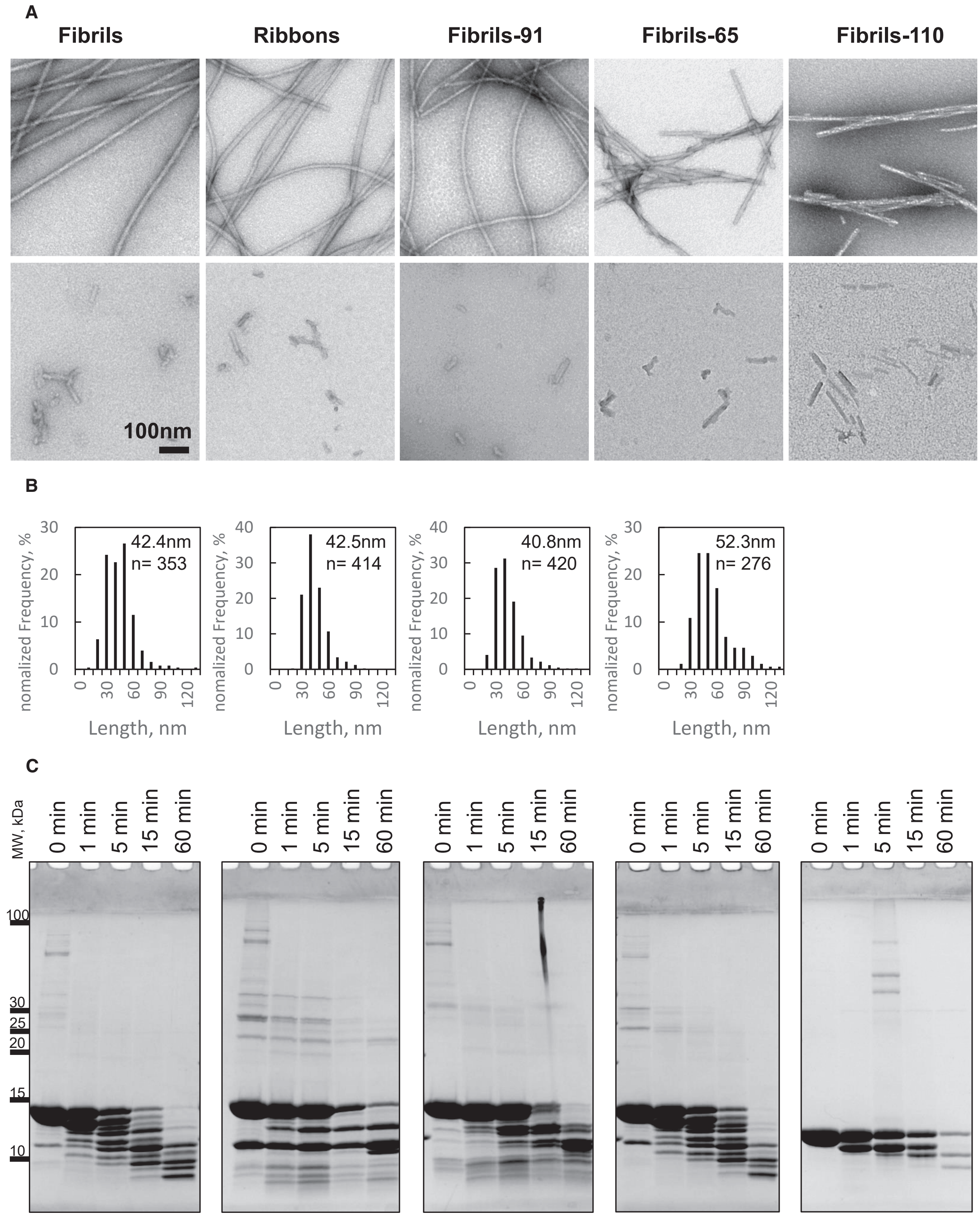

FIGURE 1 Characterization of five fibrillar α-Syn polymorphs. (*A*) Transmission electron micrographs of negatively stained α-Syn fibrillar polymorphs fibrils, ribbons, fibrils-91, fibrils-65, and fibrils-110 before (*upper lane*) and after fragmentation (*lower lane*) are shown. (*B*) Length distribution of the fragmented fibrillar polymorphs is shown. The number (n) of fibrillar assemblies the histograms were derived from is indicated. (*C*) Proteinase K degradation patterns of α-Syn (100 μM monomer concentration) polymorphs fibrils, ribbons, fibrils-91, fibrils-65, and the fibrillar form of the truncated form of α-Syn spanning residues 1–110 monitored over time on Coomassie-stained SDS-PAGE (12%) are shown. Time (min) and molecular weight markers (kDa) are shown on the top and left of each gel, respectively.

TABLE 1 Antibodies Used for Immunolabeling and Quantification

Primary Antibody	Host	Supplier	Fixation Protocol	Dilution
Organotypic Slices				
pS129-α-Syn (81A)	mouse monoclonal	Millipore MABN826		1:1200
p62/sequestosome-1	rabbit polyclonal	Proteintech 55274-1-AP	4% PFA	1:1000
NeuN (neurons)	mouse monoclonal	Millipore MAB377	4°C	1:800
Iba1	rabbit polyclonal	Wako	45 min	1:1000
Olig2	rabbit polyclonal	Millipore AB9610		1:500
Primary Neurons				
pS129-α-Syn (81A)	mouse monoclonal	Millipore MABN826		1:1200
p62/sequestosome-1	rabbit polyclonal	Abcam ab51253	4% PFA	1:1000
τ (Axons)	rabbit polyclonal	Synaptic System 314003	room temperature	1:1200
α3-NKA	mouse monoclonal	Thermo Fisher Scientific MA3-915	10 min	1:800
mGluR5	rabbit polyclonal	Millipore AB5675		1:1200
vGluT1	guinea pig polyclonal	Millipore AB5905		1:1000
GluA1-AMPA	rabbit polyclonal	Synaptic System 182003		1:1200
GluA2-AMPA	rabbit polyclonal	Synaptic System 182103	Methanol	1:1200
GluN2B-NMDA	mouse monoclonal	NeuroMab 75-101	−20°C	1:200
			10 min	

(Alexa647) frames were acquired. STORM imaging was carried out on an inverted Nikon Eclipse Ti microscope (Tokyo, Japan) equipped with a 100× oil- immersion objective (numerical aperture 1.49 with a microscope-inbuilt 1.5× lens) using an Andor iXon electron multiplying charge coupled device camera (image pixel size, 106 nm; Andor, Belfast, UK). ATTO-647N/Alexa647 were imaged using a 639 nm laser (1 kW, used at 500 mW) for a 50-ms exposure time. Single molecules were detected and rendered with a pointing accuracy of 10 nm (Gaussian radius, 10 nm) using MATLAB. All the quantifications were performed using open-source software ImageJ and Lama (25); the latter was used to compute the density-based spatial clustering of applications with noise (DBSCAN) algorithm (26). DBSCAN allows the identification of clusters in large spatial data sets by looking at the local density of points. Here, after correcting for multiple detections in consecutive frames, a "density threshold" of a minimum of 20 detections within a radial distance of 20 nm was used.

For single-particle-tracking (SPT)-STORM, neurons were exposed to Photoactivable Janelia Farm 646-labeled α-Syn polymorphs (50 nM). Imaging was performed within 10 min of exposure to study the dynamic properties of fibrillar assemblies at the membrane. These photoactivable (off to on) dyes are excellent for live-cell imaging, especially in SPT experiments, in which they enable longer observations and better localization of individual fluorescent conjugates (27). Exposure and recording were performed in Krebs recording medium (110 mM NaCl, 4 mM KCl, 1.5 mM $CaCl_2$, 1.2 mM $MgSO_4$, 25 mM $NaHCO_3$, 1 mM NaH_2PO_4, 20 mM Hepes, 10 mM glucose (pH 7.4)). Imaging was performed as recently described (19). Photoactivable Janelia Farm 646 images were acquired at 50 Hz (20 ms) using a 647 nm laser (0.5 kW, used at 200–300 mW) while pulse activating with a 405 nm laser (100 mW power, used at 2–5 mW) for 6000 frames.

Hidden Markov model

The most probable model of diffusive states was inferred by a modified version of vbSPT analysis software (28), which applies a Bayesian treatment of hidden Markov models. This approach was recently implemented in our work to track membrane proteins (19). The number of trajectories analyzed were (each experiment) fibrils: 2982, 21,202, 12,041; ribbons: 17,322, 26,941, 33,225; and fibrils-91: 11,237, 12,694, 45,426. We assume that α-Syn single molecules remain in a steady state within the short experimental observation time. This takes into account 1) binding of new molecules to the membrane, 2) cluster formation, and 3) dissociation of single molecules from clusters and targeting to endocytosis compartments. The analysis vbSPT method uses a maximal evidence criterion to determine the most probable number of diffusive states from each set of observed data (n = 3 experiments). The script was allowed to freely choose between models with one, two, or three possible states. Only the position coordinates of the molecules in two successive time points were taken into account to construct the model. Based on our previous data (19), prior values of D and dwell time were 0.1 $\mu m^2/s$ and 50 frames (1000 ms), respectively. The minimal length of trajectory was 2.

Multielectrode array recordings

Primary neuronal cultures were grown on 120-electrode multielectrode array (MEA) plates (120MEA30/10iR-ITO; MultiChannel Systems, Reutlingen, Germany) at a density of 240,000 cells/plate. Neuronal activity was sampled at 10 kHz using MultiChannel Experimenter software and MEA2100-System (MultiChannel Systems, Reutlingen, Germany). Cells were kept in their culture medium during recordings. In these conditions, cultured neurons maintain the same network activity for at least 60 min. To avoid movement-induced artifacts, recordings were started 15 min after translocation of MEAs from incubator to the recording stage. Analysis was carried out on 10-min-long sessions for each plate. Spikes, considered as point processes, were detected (±6 standard deviations) in high-pass (300 Hz)-filtered records. MEA plates were immediately put back in the incubator after recording. Channels with a mean firing rate <0.1 Hz were considered as nonspiking and discarded from further analyses. Signal processing and all analyses of neuronal activity were carried out using home-made software in MATLAB.

To compare the effect of treatments on network activity, the activity of 14 DIV cultures grown on MEA was recorded as above. After recording, cultures were let to recover at least 2 h in the incubator before application of fibrillar polymorphs. 1 week later, MEA plates were recorded again. Results are expressed as the normalized ratio of change between 14 DIV and 21 DIV to account for the inherent differences in network development between MEAs.

Graphs and statistics

Image analysis were performed on ImageJ and MATLAB. Graphs were plotted and statistics performed on GraphPad Prism software. All plots show the distribution of values as box plots, detailed within the legend. Dot plots shows the

averaged value per experiment. When both box plot and dot plot are shown, the statistical test is performed on the box plot data. A nonparametric Mann-Whitney has been performed to compare the distribution and tests whether the median of two groups are independent of each other.

RESULTS

Differential α-Syn fibrillar polymorphs binding and clustering on primary neuronal cultures

We previously demonstrated the ability of monomeric α-Syn to assemble into fibrillar polymorphs that differ through the conformation the protein adopts within the fibrils and the packing of α-Syn molecules within the fibrils under different experimental conditions (5,8,18). The resulting pure polymorphs differ in their shape on transmission electron micrographs (Fig. 1 *A*, *top row*). The polymorphs were fragmented so that they have the same average length (Fig. 1 *A*, *bottom row*, and Fig. 1 *B*). They possess distinct limited proteolytic patterns, with the exception of fibrils and fibrils-65, because they expose differently proteinase K cleavage sites (Fig. 1 *C*). These proteolytic patterns can be compared to "fingerprints," reflecting the different conformations α-Syn molecules adopt within distinct fibrillar polymorphs. They also exhibit distinct physical and pathogenic properties (4,18).

To determine to what extent the intrinsic structure of fibrillar α-Syn polymorphs affect binding to neurons, we exposed primary neurons at DIV 21 to identical concentrations of ATTO-550-labeled fibrils, ribbons, fibrils-91, fibrils-65, and fibrils-110 (50 nM) (18) for 5 or 60 min. At these time points, most of the fibrillar α-Syn remain bound to the plasma membrane (10). The cultures were next immunolabeled for Homer to identify excitatory synapses along the dendrites (Fig. 2 *A*). The images reveal striking differences. Fibrils-91 bound to neurons with a much better efficiency as compared

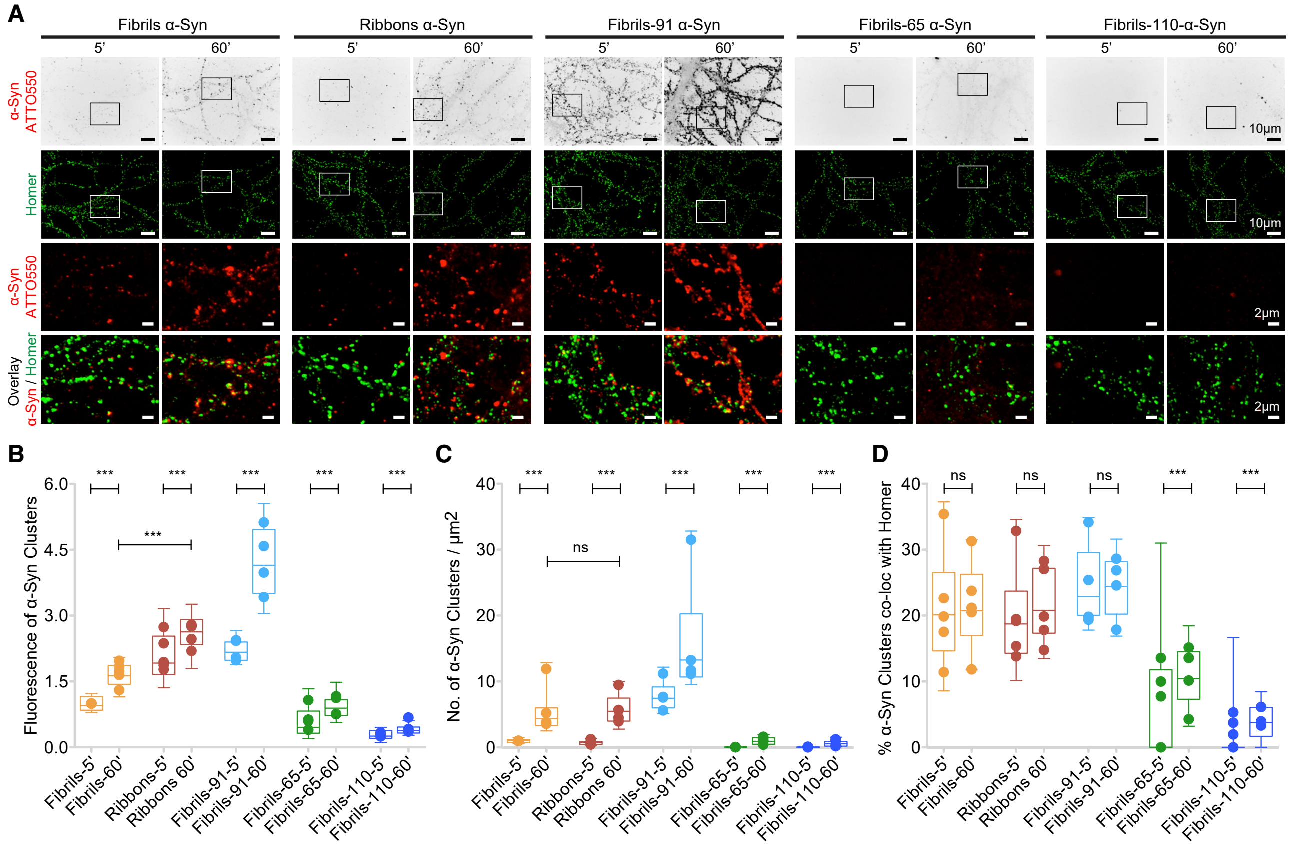

FIGURE 2 Differential fibrillar α-Syn polymorphs binding and clustering on primary neuronal cultures. (*A*) Cultured hippocampal neurons (DIV 21–24) exposed for 5 or 60 min to the fibrillar α-Syn polymorphs fibrils, ribbons, fibrils-91, fibrils-65, and fibrils-110 (50 nM) labeled with ATTO-550, followed by fixation and immunolabeling of Homer (excitatory postsynapse marker), are shown (*top two row*: *full field view*; *bottom two rows*: *boxed region*). Full-field view of ATTO-550 fluorescence is shown in grayscale (*top row*) for better visualization of neuronal morphology. The images reveal striking differences in binding. Fibrils-91 bound much more efficiently than the ribbons and fibrils polymorphs. Fibrils-65 and fibrils-110 exhibited weak binding. All three polymorphs exhibited significant colocalization with Homer (*bottom row*). (*B–D*) Quantification of size (*B*), fluorescence intensity of fibrillar ATTO-550-α-Syn polymorphs clusters, density (*C*), number of fibrillar ATTO-550-α-Syn polymorphs clusters per μm^2, and synaptic colocalization (*D*) of fibrillar ATTO-550-α-Syn polymorph clusters obtained after thresholding are shown (see Material and Methods). (*D*) The proportion of synaptic fibrillar α-Syn polymorph clusters were similar for all polymorphs except fibrils-65 and fibrils-110. The box plot shows median, interquartile range, and 10–90% distribution. A Mann-Whitney test was performed to compare the distribution between 5 and 60 min; 60–75 images from four to five independent experiments. ***$p < 0.001$; ns, not significant. Dot plot shows the averaged value per experiment.

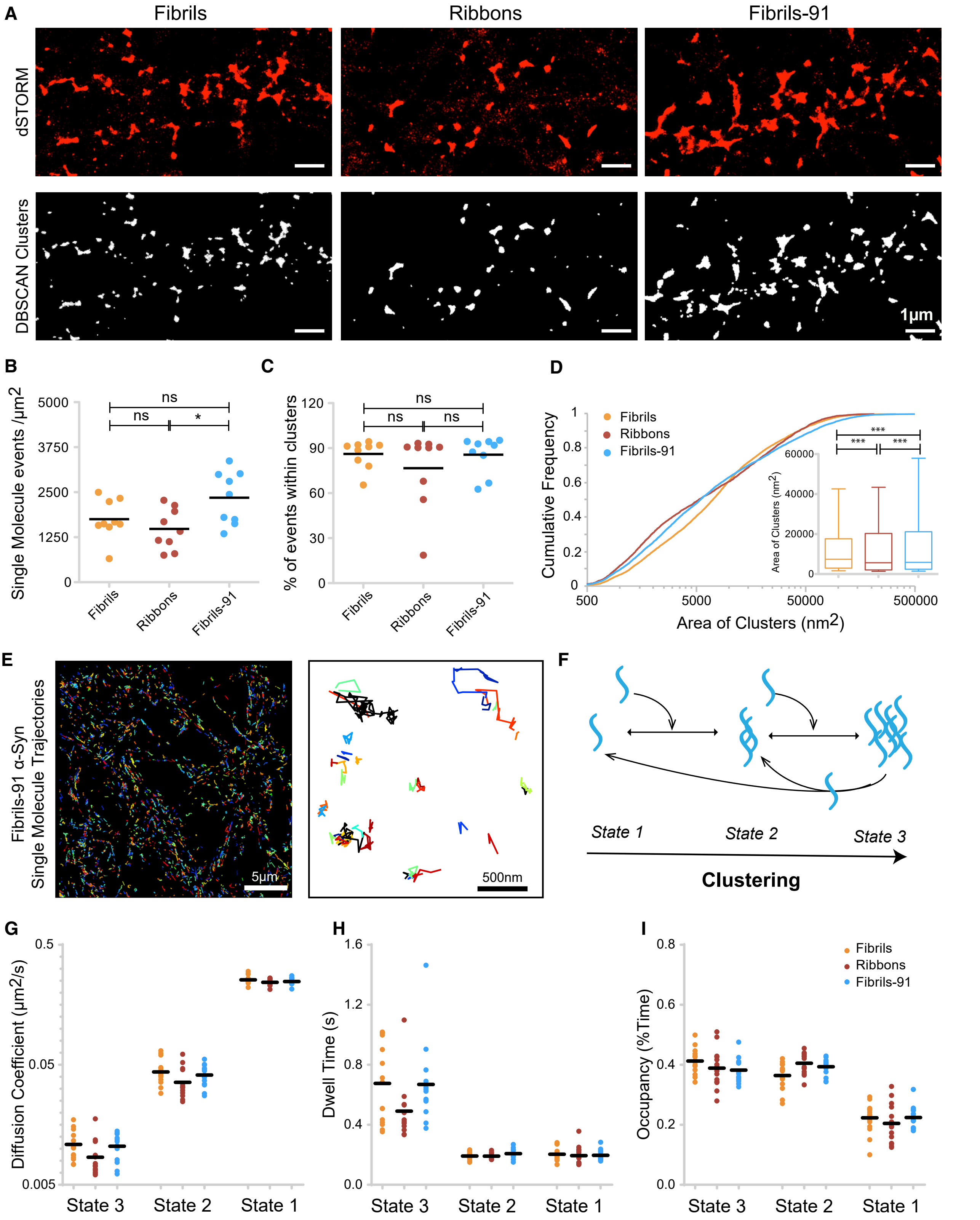

FIGURE 3 Nanoscopic properties of the clusters fibrillar α-Syn polymorphs form on primary neuron plasma membrane. (*A*) Super-resolution images rendered with a pixel size of 10 nm (*red*, *top row*) show the binding and clustering of fibrillar α-Syn-ATTO-647N polymorphs on neurons after 60 min exposure to the fibrillar polymorphs fibrils, ribbons, and fibrils-91 (50 nM). Bottom row (*white*) shows fibrillar α-Syn-ATTO-647N clusters identified through the DBSCAN analysis (see Material and Methods). (*B*) The density (detections per μm^2) of single-molecule events on neurons (*clustered* and *nonclustered*) in

(legend continued on next page)

to the polymorph fibrils (threefold difference; Fig. 2 *A*, *rows 1* and *3*). In contrast, fibrils-65 and fibrils-110 bound to neurons with much less efficiency than the α-Syn polymorph fibrils. Ribbons bound to neurons with an efficiency significantly higher than that of fibrils but lower than that of fibrils-91. The "fluorescence of α-Syn cluster," indicative of the size of the cluster, and the "number of α-Syn clusters per μm^2," characteristic of density, were quantified (Fig. 2, *B* and *C*, respectively). The size of α-Syn ribbon clusters was larger than that of the fibrillar polymorph fibrils (Fig. 2 *B*), whereas both exhibited similar density (Fig. 2 *C*). In contrast, the size and density of α-Syn fibrils-91 clusters were significantly larger than those of α-Syn polymorph fibrils. Both the binding efficiency and the density of the cluster fibrils-65 and fibrils-110 formed on neurons were significantly lower than those of the polymorph fibrils.

In the primary hippocampal neuronal cultures, 80–85% synapses are excitatory. We therefore assessed the partial colocalization and apposition of α-Syn fibrillar polymorphs with the excitatory synapse marker Homer. Nearly 15–25% of the clusters of the fibrillar α-Syn polymorphs that bound efficiently to neurons (e.g., fibrils, ribbons, and fibrils-91) colocalized with excitatory synapses (Fig. 2 *D*), independent of the exposure time (5 or 60 min). The figure was smaller—0–12% colocalization with Homer—for the two fibrillar polymorphs (fibrils-65 and fibrils-110) that bound significantly less efficiently to neurons. This is in line with our previous study, in which we reported that α-Syn fibrils form clusters at both synaptic and extrasynaptic neurons and also on axon and dendrites (Fig. S1; (10,29)). Our observations undoubtedly demonstrate that distinct α-Syn fibrillar polymorphs bind to and cluster on neuron plasma membrane to different extents. We therefore conclude from these observations that the amino acid stretches exposed at the surfaces of distinct pathogenic α-Syn fibrillar assemblies define the efficiency with which they bind to neuronal plasma membranes.

Nanoscopic properties of fibrillar α-Syn fibrillar polymorph clusters on neuronal membrane

Confocal imaging and threshold-based analysis (Fig. 2) are biased toward larger clusters and provide no information about diffused single molecules (nonclustered) and molecules forming nanoclusters. These limitations are solved by the use of super-resolution imaging. We therefore performed STORM on fixed neurons exposed to the different fibrillar α-Syn polymorphs (50 nM, 60 min) labeled with ATTO-647N dye, as in a previous study (10). Representative rendered images of the fibrillar polymorphs fibrils, fibrils-91, and ribbons with a pointing accuracy of 10 nm are shown (Fig. 3 *A*, *upper row*). DBSCAN, a point-detection-based clustering algorithm, was employed to confine analysis to authentic clusters (25,26) (Fig. 3 *A*, *bottom row*). The densities of the polymorphs fibrils and ribbons bound to neurons (single-molecule detections per μm^2) (Fig. 3 *B*) were similar but smaller (1.5-fold lower) than the density observed for fibrils-91 (Fig. 3 *B*). Despite this, as much as 90% of all single-molecule detection events for all three α-Syn fibrillar polymorphs were localized within the clusters identified by DBSCAN (Fig. 3 *C*). These observations clearly indicate that α-Syn fibrillar polymorphs have a high propensity to form clusters on the plane of the plasma membrane. The cumulative distribution of area of all clusters (n = fibrils: 7681, ribbons: 4600, fibrils-91: 8672) shows a wide distribution. The distribution reveals significant differences between the clusters the three fibrillar polymorphs form (Fig. 3 *D*). Compared to fibrils, the polymorph ribbons populates small sized clusters (area < 5000 nm^2). The polymorph fibrils-91 populates very large clusters ($>$200,000 nm^2) that are observed neither for fibrils nor ribbons.

We further performed SPT of α-Syn fibrillar polymorphs on the plasma membrane of live neurons using STORM (SPT-STORM). For this, preformed α-Syn fibrillar polymorphs were labeled using Photoactivable Janelia Farm 647 dye (27). This powerful imaging approach allowed us to track the dynamic behavior of thousands of α-Syn single fibrillar particles on neuron membranes and quantify their dynamics before cluster formation. The measurements were performed within 10 min after exposure of neurons to α-Syn polymorphs (50 nM) to track specifically membrane-bound assemblies. Representative images (Fig. 3 *E*) show single-particle trajectories of α-Syn fibrils-91 at two magnifications. By using variational Bayesian treatment of

(*A*) is given, showing differential binding of the distinct fibrillar α-Syn polymorphs. (*C*) The proportion of single-molecule detections within clusters is given, showing predominantly clustered (90%) binding of the distinct fibrillar α-Syn-ATTO-647N polymorphs. (*D*) Measurement of area of clusters (μm^2) is shown as a cumulative distribution plot and box plot (*inset*, n = fibrils: 7681, ribbons: 4600, fibrils-91: 8672). The distribution shows that the fibrillar polymorph fibrils-91 populates clusters $>$200,000 nm^2. The cumulative plot shows that the polymorph ribbons populates the smallest clusters ($<$500 nm^2). (*E*) A representative example is given, showing single-molecule trajectories (*colored*) obtained using SPT-STORM of the α-Syn-Photoactivable-646 fibrils-91 polymorph at two magnifications. Trajectories were analyzed by Bayesian treatment of hidden Markov models (see Materials and Methods), which revealed three diffusive states of α-Syn polymorphs. (*F*) State 1 represents free, fast-diffusing molecules; state 2 exhibits intermediate diffusion velocity, representing small complexes; and state 3 represents a clustered fraction with very slow diffusion velocity. (*G–I*) Plots showing averaged diffusion coefficient values (*G*) and dwell time within each diffusive state (*H*) and occupancy (*I*) for each polymorph are given. Note the ribbons polymorph exhibits characteristic different diffusive behavior. Each dot represents the averaged value of thousands of trajectories for a given imaging field. The dot plots in (*B*) and (*C*) represent averaged value per image, n = 9 cells from three independent experiments. The box plot in (*D*) shows median, interquartile range, and 10–90% distribution. The dot plots in (*G*) and (*H*) show averaged value per recording (thousands of trajectories) from three independent experiments. A Mann-Whitney test was performed. $^*p < 0.05$, $^{***}p < 0.001$; ns, not significant. Scale bars, 1 μm.

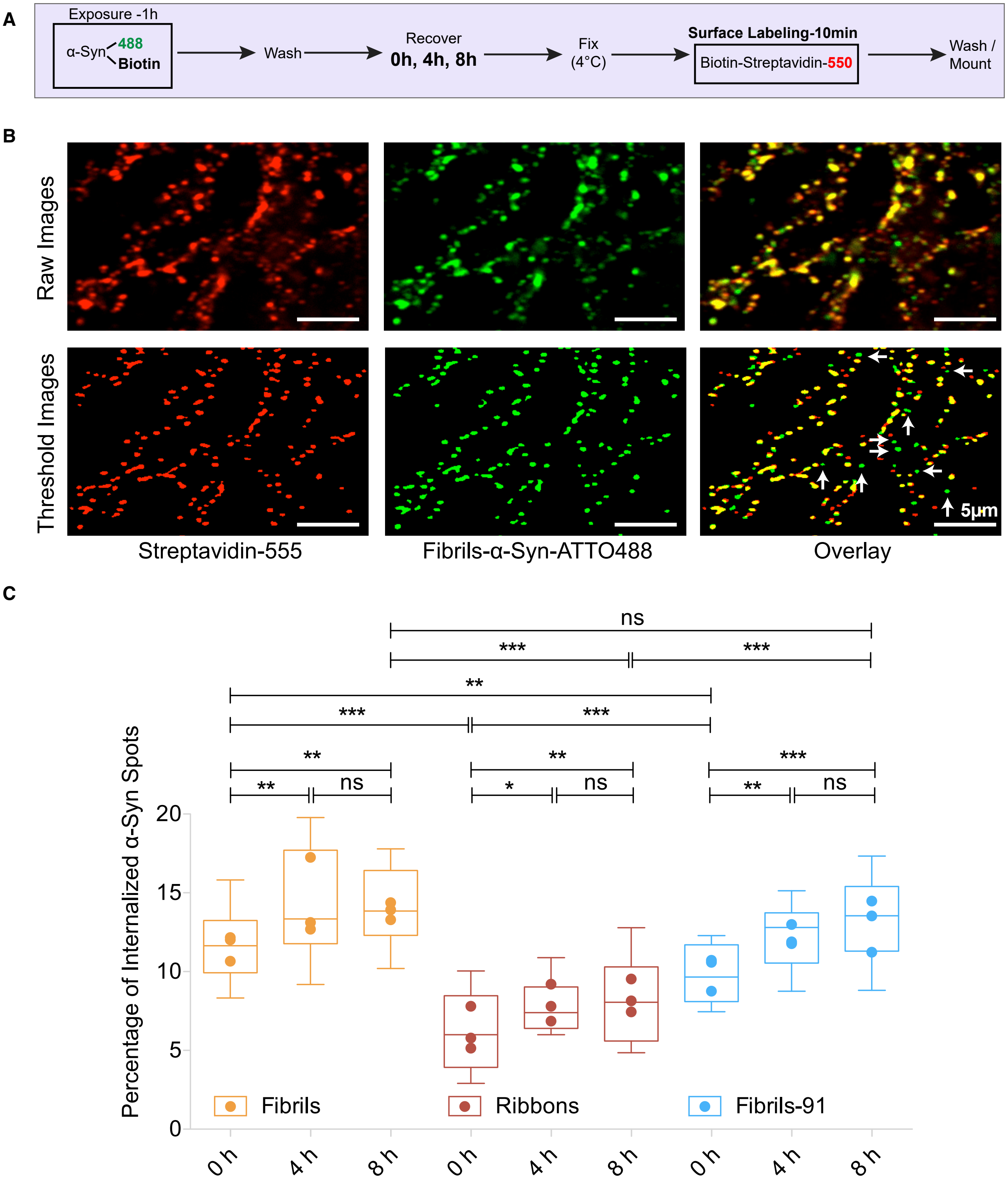

FIGURE 4 Assessment of time-dependent endocytosis of α-Syn fibrillar polymorphs. (*A*) A schematic representation of the experimental setup to measure the initial endocytosis of fibrillar α-Syn polymorphs is given. Neurons were exposed (50 nM, 60 min) to ATTO-488 + biotin-labeled α-Syn. Unbound assemblies were next washed away. Cells were fixed 0, 4, or 8 h after exposure to fibrillar α-Syn polymorphs (*green*), and α-Syn clusters remaining on the cell surface were labeled with streptavidin550 (*red*). (*B*) Representative raw (*top panel*) and thresholded (*bottom panel*) images for α-Syn fibrillar polymorph fibrils labeled with ATTO-488 (*green*) and biotin are shown. α-Syn fibrils remaining on the cell surface were labeled with streptavidin550 (*red*). The majority

(legend continued on next page)

hidden Markov models (28) that combine information from thousands of short single-particle trajectories, three diffusive states of α-Syn were extracted with confidence. Particles in state 1 represent fast-diffusing, most likely single fibrils; particles in state 2 exhibit intermediate diffusion velocity and diffuse as small complexes; particles in state 3 are characterized by slow diffusion velocity and represent clustered α-Syn fibrillar assemblies (Fig. 3, *F* and *G*). Comparison of the diffusion coefficients indicate that the polymorphs fibrils and fibrils-91, but not ribbons, diffuse identically in all three states. This indicates that the polymorphs ribbons and fibrils/fibrils-91 interact differentially with membrane components. Single molecules can exchange between diffusive states (Fig. 3 *F*); therefore, we quantified the dwell time of a single molecule in a given diffusive state. All α-Syn polymorphs exhibit a higher dwell time in state 3 (i.e., in the clustered state (Fig. 3 *H*)) compared to nonclustered states (state 1 and 2). This validates the observation that α-Syn has a high propensity to form clusters (Fig. 3 *B*). Interestingly, in diffusive state 3, ribbons exhibit lower dwell-time values compared to fibrils/fibrils-91 (Fig. 3 *H*). This strongly suggests that the clusters ribbons form are less stable than those fibrils and fibrils-91 yield. The occupancy time (i.e., the time spent by single particles in each diffusive state) is plotted (Fig. 3 *I*). The three polymorphs spend less time (20%) in state 1 (as single particles) compared to states 2 and 3 (in a larger complex), indicating cluster formation after diffusion.

Time-dependent endocytosis of fibrillar assemblies

After binding to the membrane, fibrillar α-Syn polymorphs are internalized (30,31). We quantified the rate of initial endocytosis of distinct fibrillar α-Syn polymorphs because this is key for subsequent seeding within neuronal cells. Primary mature neurons at DIV 21 were exposed to fibrillar α-Syn polymorphs for 1 h (50 nM, dual labeled with biotin and ATTO-488) (Fig. 4 *A*), followed by removal of unbound assemblies. Cells were fixed immediately (0 h condition) or after 4 or 8 h of exposure. Whereas total (surface + endocytosed) α-Syn polymorphs were detected based on ATTO-488 fluorescence, those at the cell surface were detected using streptavidin550, which binds to biotin. ATTO-488 spots that did not colocalize with streptavidin550 spots depicted endocytosed fibrillar α-Syn. As shown in the representative example (Fig. 4 *B*), a small proportion of fibrillar α-Syn polymorphs were endocytosed (*green only spots*) within this time frame. Quantification showed a small but significant time-dependent increase in the number of internalized fibrillar α-Syn spots for all three polymorphs (Fig. 4 *C*). Notably, for all fibrillar polymorphs, 5–15% spots were found endocytosed; the remaining fraction was localized at the plasma membrane. This contrasts with fibrillar α-Syn polymorph differential binding and suggests that the rate of endocytosis of various α-Syn polymorphs of similar length is independent from their intrinsic structures in agreement with previous observations (30).

Differential seeding by fibrillar α-Syn polymorphs in primary neuronal cultures

To determine whether the differential binding of distinct fibrillar α-Syn polymorphs reflects in their seeding propensity, we assessed quantitatively the aggregation of endogenous α-Syn using mature primary neuronal cultures. Hippocampal neurons at DIV 14, when synapses and spines (32) are formed, were exposed to the three fibrillar α-Syn polymorphs that bind best to neurons (i.e., fibrils, ribbons, and fibrils-91) for 15 min (250 nM). At DIV 21, cells were fixed and immunolabeled for pS129-α-Syn using 81A antibody (Fig. 5 *A*). A few processes (nearly three to five on an 18-mm coverslip with 100,000 cells plated) were pS129-α-Syn positive after exposure of neurons to the polymorphs fibrils (Fig. 5, *B* and *C*). The number of pS129-α-Syn-positive processes was significantly larger upon exposure of neurons to the polymorph ribbons and even larger upon exposure to the polymorph fibrils-91 (Fig. 5, *B* and *C*). The pS129-α-Syn we detected is of endogenous nature because similar results were obtained with exogenous fibrillar polymorphs made of an α-Syn version (α-Syn S129A) that cannot be phosphorylated on serine residue 129 (Fig. S2). As expected, pS129-α-Syn immunoreactivities were located within axons (Fig. 5 *D*), where endogenous α-Syn is expressed (33). The autophagosome marker p62/sequestosome-1 colocalized with pS129-α-Syn bundles within the cell body (Fig. 5 *E*), but not with those in the axons (Fig. 5 *E*, *arrows*). pS129-α-Syn bundles within the cell body also colocalized with ubiquitin (Fig. 5 *F*). To determine whether seeding affects the integrity of neurons in primary cultures, neurons were stained with anti-Homer antibodies, and the density of synapses was quantified. A slight reduction in Homer-positive synapses was observed in neurons where endogenous α-Syn was seeded by fibrils-91 (Fig. S3 *A*).

1% sarkosyl extract was prepared from seeded neurons and analyzed on 12% SDS-PAGE gel without a stacking layer. Western blot analysis revealed pS129-α-Syn immunoreactivity for ribbons and fibrils-91 polymorphs (Fig. 5 *G*, *left*). Mouse-specific α-Syn detected monomeric (all

of α-Syn clusters were on the cell surface (*overlay*, *yellow*). Internalized fibrillar α-Syn spots (*green* only) were quantified and plotted in (*C*). (*C*) A time-dependent increase in fibrillar α-Syn polymorph endocytosis is observed. The box plot shows median, interquartile range, and 10–90% distribution. A Mann-Whitney test was performed to compare the distributions; 30 images from three independent experiments. ***$p < 0.001$, **$p < 0.01$, *$p < 0.05$; ns, not significant. Dot plot shows the averaged value per experiment.

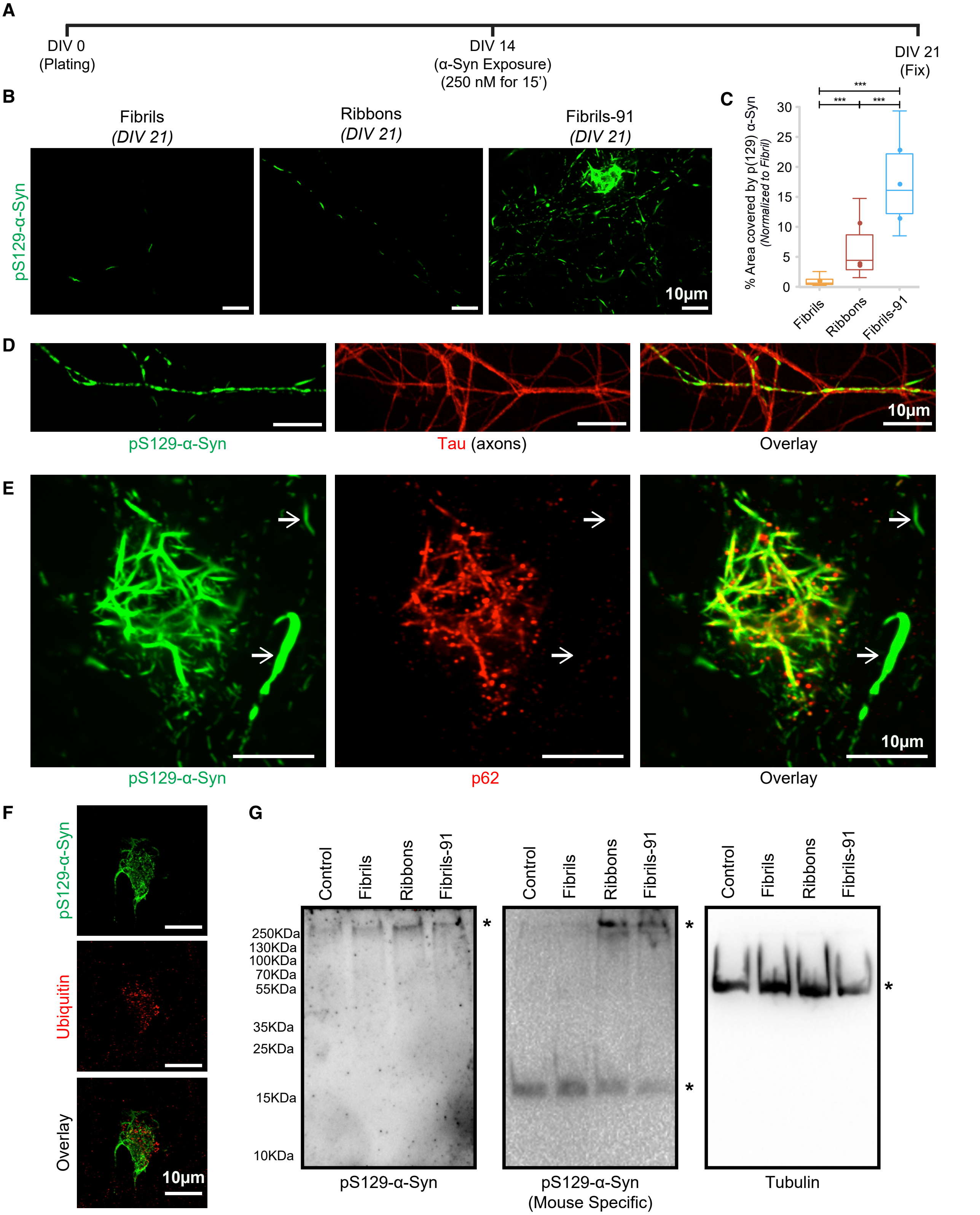

FIGURE 5 Differential seeding by fibrillar α-Syn polymorphs in primary neuronal cultures. (*A*) A schematic representation of the protocol used to assess the seeding of endogenous α-Syn by exogenous fibrillar α-Syn polymorphs fibrils, ribbons, and fibrils-91 is shown. Primary mature hippocampal cultured neurons (prepared from WT C57BL6J mice) were exposed to fibrillar α-Syn polymorphs (250 nM, 15 min in fresh culture medium) at DIV 14. After extensive washing, the cells were transferred back to the original culture medium. Neurons were fixed at DIV 21 and immunolabeled for pS129-α-Syn. (*B*) Seeded

(legend continued on next page)

conditions) as well as aggregated α-Syn (ribbons and fibrils-91 conditions) (Fig. 5 *G*, *middle*). Surprisingly, the immunoreactivity for fibrils-91 was not higher than that of ribbons as anticipated. This could be due to differential turnover of pS129-α-Syn aggregates seeded by fibrils, fibrils-91, and ribbons. We conclude from these observations that α-Syn fibrillar polymorphs binding to neurons is key for seeding endogenous α-Syn. Indeed, the fibrillar polymorph that bound the best within 1 h (Fig. 2) seeded the most after 1 week (Fig. 5).

Seeded pS129-α-Syn aggregates are composed of multiple elongated intertwined structures

We performed STORM imaging to visualize seeded pS129-α-Syn at higher resolution to determine whether they exhibit different macromolecular characteristics. Aggregated axonal pS129-α-Syn exhibited multiple microscopic elongated structures irrespective of the fibrillar polymorphs the neurons were exposed to (Fig. 6, *A*–*C*). The thickness of the elongated structures was within the range 30–40 nm. We next assessed the time course of these elongated structures formation using the polymorph fibrils-91, which yields the largest amount of pathology. Aggregated axonal pS129-α-Syn formation was imaged by STORM after exposure of primary neurons to fibrillar polymorphs for 2, 3, and 6 days (Fig. 6 *D*). The elongated structures appear as early as 2 days postexposure. They are fewer in number at 2 (Fig. 6 *D*, *top panel*) than 3 (Fig. 6 *D*, *middle panel*) and 6 (Fig. 6 *D*, *bottom panel*) days postexposure. Quantification confirms that the number of pS129-α-Syn molecules (detection events) within the aggregates (Fig. 6 *E*) and the area occupied by aggregates (Fig. 6 *F*) increases from day 2 to 6. STORM imaging also revealed a time-dependent thickening of those elongated structures with the appearance of bundles (Fig. 6 *D*, *bottom row*).

Differential α-Syn fibrillar polymorphs binding and seeding in organotypic slice cultures

To determine whether fibrillar α-Syn polymorphs binding to neurons and seeding in vivo mirrors the observations we report using primary neuronal culture, we developed an ex vivo organotypic hippocampal slices model. Slices maintained for 2 weeks after plating were exposed for 15 min to the fibrillar polymorphs fibrils, ribbons, and fibrils-91 labeled with ATTO-550 (0.75 μM). The different fibrillar α-Syn polymorphs bound to all the available surface of organotypic slices (Fig. 7 *A*). As for primary neuronal cultures, the polymorph fibrils bound the least, whereas the polymorph fibrils-91 bound the most (Fig. 7, *A* and *B*). Seeding of endogenous α-Syn by the different fibrillar polymorphs was next assessed. The slices were fixed 4, 7, or 14 days postexposure to exogenous fibrillar α-Syn polymorphs (Fig. 7, *C*–*E*), and aggregated α-Syn was detected using pS129-α-Syn immunolabeling with the mouse monoclonal antibody 81A (Fig. 7, *green*). Processes immunopositive for pS129-α-Syn were visible as early as 4 days after exposure to the polymorph fibrils-91, but not after exposure to the other fibrillar polymorphs (Fig. 7, *D* and *E*). At day 7, few pS129-α-Syn-positive processes were detected in slices exposed to the polymorphs fibrils and ribbons (Fig. 7, *D* and *E*). By day 14, slices exposed to ribbons exhibited a larger number of pS129-α-Syn-positive processes than those exposed to fibrils, albeit fewer than those exposed to fibrils-91 (Fig. 7, *D* and *E*). No hippocampal region specificity was observed. As for the primary neuronal cultures, pS129-α-Syn aggregates are made from the endogenous α-Syn because similar results were obtained with exogenous fibrillar polymorphs made of an α-Syn version (α-Syn S129A) that cannot be phosphorylated on serine residue 129 (Fig. S4). The residual fluorescence of exogenous fibrillar α-Syn polymorphs were also assessed on days 4, 7, and 14 (Fig. 7 *F*). A time-dependent decrease, demonstrating clearance and degradation of exogenous fibrillar α-Syn polymorphs, was observed.

Loosely packed aggregated bundles of endogenous pS129-α-Syn (Fig. 7, *G* and *H*, green) were observed in neuron cell bodies (Fig. 7 *G*, *gray*) either frequently or occasionally upon exposure of the slices to the fibrillar polymorphs fibrils-91 or ribbons, respectively. The extent of pS129-α-Syn aggregation was very low (Fig. 7 *E*) in slices exposed to the polymorph fibrils, and no aggregated bundles were seen in the experimental timeframe. These structures stained positive for autophagosome marker p62/sequestosome-1 (Fig. 7 *H*, *red*). Notably, p62 only colocalized with cytosolic bundled aggregates, but not individual processes (Fig. 7 *H*, *arrows*), as observed in primary cultures (Fig. 5 *E*).

endogenous α-Syn aggregation after exposure of primary neuronal cultures to the fibrillar α-Syn polymorphs fibrils, ribbons, and fibrils-91 is shown, imaged using the monoclonal anti-pS129-α-Syn antibody 81A. (*C*) Quantification of the area occupied by aggregated pS129-α-Syn after exposure to the different fibrillar α-Syn polymorphs is shown. The box plot shows median, interquartile range, and 10–90% distribution. Number of images (n): fibrils, 30; ribbons, 34; fibrils-91, 34, from three independent experiments. A Mann-Whitney test was performed, ***$p < 0.001$. The dot plot shows the averaged value per experiment. (*D*) Seeded endogenous pS129-α-Syn (*green*) within axons (*red*, labeled with anti-τ antibody) is shown. (*E*) pS129-α-Syn bundles (*green*) are stained by the autophagy marker p62 (*red*) in the cell body, but not in the processes (*arrows*). (*F*) pS129-Syn bundles (*green*) are stained by ubiquitin (*red*). (*G*) 1% sarkosyl extract from unseeded or polymorph-seeded neurons is shown. Western blots after migration of extracts on SDS-PAGE gels without stacking layer and probing for pS129-α-Syn (*left*), endogenous mouse α-Syn (*middle*), and tubulin (*right*) are shown. * represents the correct detected bands. Aggregated pS129-α-Syn can be detected in ribbons- and fibrils-91-seeded neurons (*left* and *middle*). Soluble endogenous mouse α-Syn is detected in all conditions (*middle*).

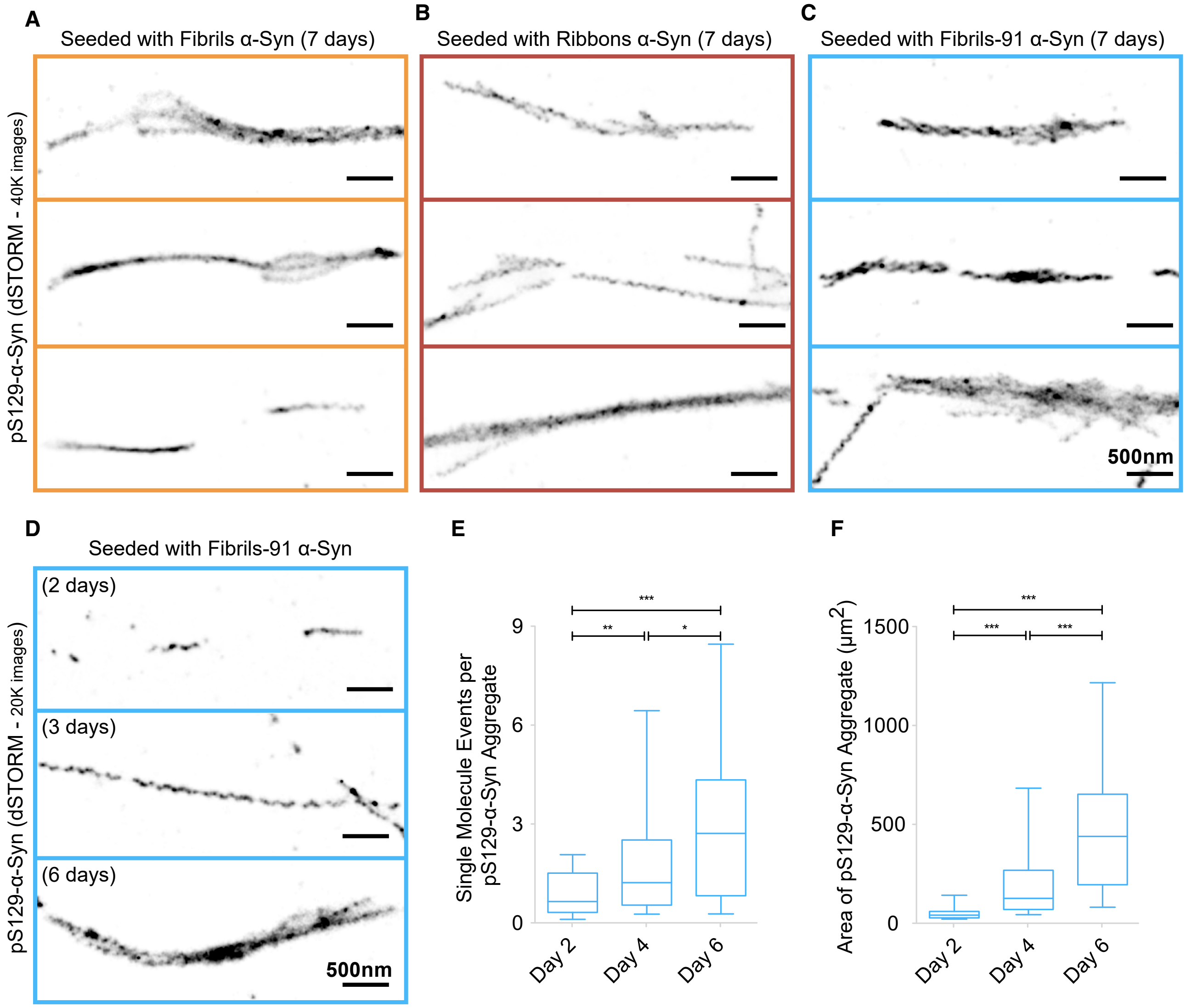

FIGURE 6 Seeded pS129-α-Syn are composed of multiple intertwined elongated structures. (*A–C*) Super-resolution STORM imaging of endogenous pS129-α-Syn was performed at DIV 21 after exposure of primary neuronal cultures to the fibrillar α-Syn polymorphs fibrils, ribbons, and fibrils-91 (250 nM) for 15 min on DIV 14. Single-molecule detections for 40,000 frames, rendered with a pixel size of 10 nm, are shown for fibrils (*A*), ribbons (*B*), and fibrils-91 (*C*). In all three cases, elongated structures that seem to intertwine into larger bundles are seen. (*D*) The time course of elongated and intertwined pS129-α-Syn structure formation imaged by STORM after exposure of primary neurons to α-Syn fibrillar polymorph fibrils-91 after 2, 3, and 6 days is shown. (*E* and *F*) Quantitative analysis of the amount of elongated and intertwined pS129-α-Syn structures forming in primary neurons exposed to fibrillar α-Syn polymorphs (250 nM for 15 min on DIV 14) as a function of time is shown. Detection events within (*E*) and area occupied by (*F*) pS129-α-Syn aggregates are shown. The box plot shows median, interquartile range, and 10–90% distribution. A Mann-Whitney test was performed to compare the distributions; n is number of pS129-α-Syn aggregates analyzed: 40 for fibrils, 52 for ribbons, 56 for fibrils-91. ***$p < 0.001$, **$p < 0.01$, *$p < 0.05$. Scale bars, 500 nm.

The dependence of seeding on the concentration of exogenous fibrillar α-Syn polymorphs was next assessed. For all three fibrillar polymorphs, a significant increase in the amount of pS129-α-Syn labeling was observed upon increasing seed concentration by twofold (Fig. 7 *I*). The increase in the median values was 100.5, 621.2, and 73.4% for the fibrillar polymorphs fibrils, ribbons, and fibrils-91, respectively (Fig. 7 *J*).

To determine whether seeding affects neuronal integrity within the organotypic slices, the slices were stained with anti-Homer antibodies, and the density of synapses was quantified after seeding. No reduction in Homer-positive synapses was observed (Fig. S3 *B*). Additionally, no observable difference in neuronal labeling was observed 1 week after seeding with the different fibrillar polymorphs (Fig. S5). We conclude from these observations that distinct fibrillar α-Syn polymorphs bind and trigger the aggregation of endogenous α-Syn to different extents ex vivo. We further conclude that they do so in a concentration-dependent manner within the concentration range we explored.

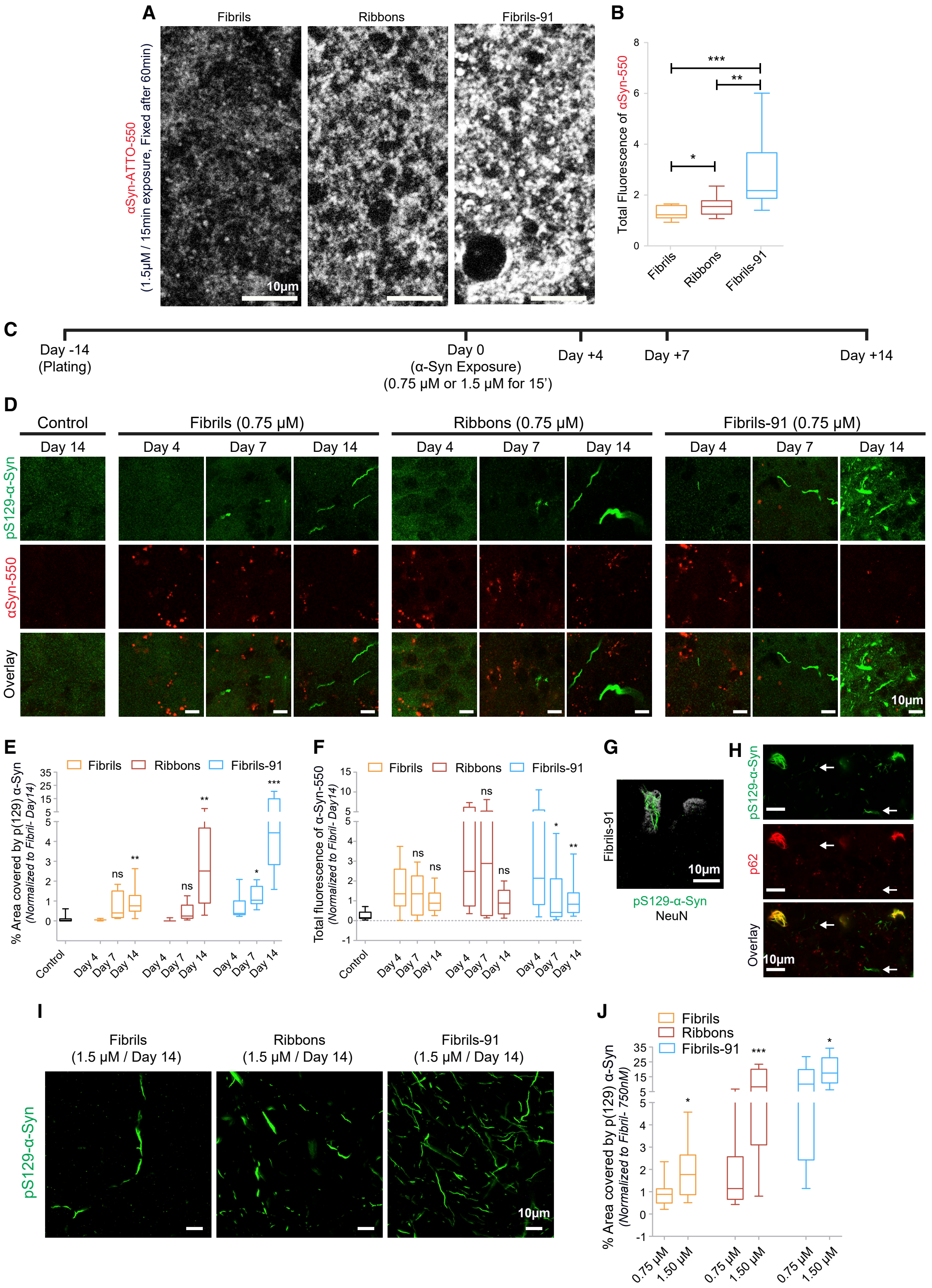

FIGURE 7 Differential α-Syn polymorphs binding and seeding in organotypic slice cultures. (*A* and *B*) Hippocampal organotypic slices maintained for 14 days in culture (day 0) were exposed to 1.5 μM ATTO-550-labeled fibrillar α-Syn polymorphs fibrils, ribbons, and fibrils-91 for 15 min, washed extensively, and transferred to a fresh slice culture medium for 1 h before fixation. (*A*) Representative confocal images are shown for the fibrillar α-Syn polymorphs fibrils, ribbons, and fibrils-91. (*B*) Quantification of fibrillar ATTO-550-α-Syn-polymorph fluorescence intensity is shown. The box plot shows median,

(legend continued on next page)

We also assessed the localization of p129-α-Syn relative to oligodendrocytes and the impact of microglia depletion from the organotypic slices on p129-α-Syn deposits. We found no p129-α-Syn deposits in Olig2-positive cells for slices exposed to fibrils and fibrils-91 polymorphs. For ribbons, we did observe occasional p129-α-Syn reactivity within Olig2-positive oligodendrocytes (Fig. S6). Because the exposed surfaces of organotypic slices are known to be enriched in microglia, we completely depleted those cells with colony-stimulating factor 1 receptor inhibitor, PLX3397 (34). This did not alter p129-α-Syn immunoreactivity, demonstrating that microglia do not affect seeding in neurons in our setup (Fig. S7).

Differential redistribution of synaptic α3-NKA/GluA2-AMPA/GluN2B-NMDA in primary neurons exposed to α-Syn fibrillar polymorphs

We next assessed the distribution of key synaptic components in seeded neurons by immunohistochemistry. Exposure was performed on DIV 14 for 15 min, and the cells were fixed on DIV 21 as above. No synaptic morphological changes were observed within the experimental time frame we used in agreement with previous observations (10). Dual detection was performed for α3-NKA (Fig. 8, *A* and *B*), GluA1-subunit of AMPA receptors (Fig. 8, *F* and *G*), GluA2-subunit of AMPA receptors (Fig. 8, *C* and *D*), GluN2B-subunit of NMDA receptors (Fig. 8, *E* and *F*), or metabotropic glutamate receptor 5 (mGluR5) (Fig. 8, *H* and *I*), along with Homer or PSD95, to identify excitatory synapses. Increased α3-NKA, GluA2-AMPA, and GluN2B-NMDA, but not GluA1-AMPA receptor or mGluR5, at synapses was observed for neurons exposed to the α-Syn fibrillar polymorph fibrils (Fig. 8). Neurons exposed to α-Syn polymorph fibrils-91 exhibited increased synaptic clustering of α3-NKA, but not GluA2-AMPA, GluN2B-NMDA, or GluA1-AMPA receptors or mGluR5 (Fig. 8). In contrast, exposure of neurons to the α-Syn fibrillar polymorph ribbons did not lead to a change in the synaptic distribution of any of the proteins or receptors we examined (Fig. 9). Our measurements demonstrate that the α-Syn fibrillar polymorphs fibrils and fibrils-91, but not ribbons, alter excitatory synaptic receptors composition. We conclude from these observations that distinct α-Syn fibrillar polymorphs differentially redistribute key synaptic components in seeded neurons, likely because of homeostatic dysregulation.

Alteration in network activity in primary neurons seeded with fibrillar α-Syn polymorphs

We next assessed the impact of fibrillar α-Syn polymorphs seeding on spontaneous neuronal activity using MEA recordings. Primary neurons were grown on 120-electrode MEA plates, and their activity was sampled at 10 kHz. To account for the inherent differences in network development between cultures, we recorded spontaneous neuronal activity on DIV 14 (2 h before exposure to α-Syn fibrillar polymorphs) and on DIV 21 (1 week after exposure). Raster plots show the network activity in each MEA in which each row represents the spiking activity around individual electrodes (Fig. 9, *A–D*). As evident from the raster plots, the most significant decrease in neuronal spiking rate was observed in fibrils-seeded neurons (Fig. 9, *B* and *E*). Neurons seeded with the polymorphs ribbons and fibrils-91 exhibit no or weak reduction in neuronal activity (Fig. 9). This observation, together with the finding that neurons exposed to the polymorph fibrils exhibit the lowest pS129-α-Syn load (Fig. 5 *C*) and the strongest redistribution of synaptic receptor (Fig. 8, *B*, *D*, and *F*), suggests that neuronal network activity is most affected by the polymorph fibrils.

DISCUSSION

We and others previously showed that megadalton fibrillar α-Syn assemblies propagate from cell to cell (4,35–39).

interquartile range, and 10–90% distribution. A Mann-Whitney test was performed. Number of images analyzed (n): 16 for fibrils, 16 for ribbons, and 12 for fibrils-91 from three independent experiments. $^*p < 0.05$, $^{**}p < 0.01$, $^{***}p < 0.001$. Scale bars, 5 μm. (*C*) A schematic representation of the protocol used to assess the seeding of endogenous α-Syn by exogenous fibrillar α-Syn polymorphs fibrils, ribbons, and fibrils-91 is given. Hippocampal organotypic slices maintained for 14 days in culture (day 0) were exposed to 0.75 or 1.5 μM ATTO-550-labeled fibrillar α-Syn polymorphs for 15 min, washed extensively, and transferred to a fresh slice culture medium. Slices were fixed at different days and stored at 4°C until the end of the experiment, when immunohistochemistry for pS129-α-Syn was performed. (*D–F*) Representative images (*D*) showing seeded endogenous pS129-α-Syn labeled with the antibody 81A (*green*, *top row*), exogenous-α-Syn-ATTO-550 (*red*, applied at 0.75 μM, *middle row*), and overlaid channels (*bottom row*) after exposure of hippocampal organotypic slices to fibrillar α-Syn polymorphs fibrils, ribbons, and fibrils-91 are shown. The percentage of area occupied by pS129-α-Syn was plotted in (*E*), and the total fluorescence of exogenous-α-Syn-ATTO-550 was plotted in (*F*). The box plot shows median, interquartile range, and 10–90% distribution. A Mann-Whitney test was performed to compare the difference from day 4 for each polymorph; the number of images (n) acquired with inclusions from three to five experiments (*left* to *right*) was 10, 3, 4, 29, 3, 5, 37, 9, 12, and 22. $^*p < 0.05$, $^{**}p < 0.0.01$, $^{***}p < 0.001$; ns, not significant. (*G*) Aggregated bundles of endogenous pS129-α-Syn structures (*green*) within the neuronal cell body (*gray*, labeled with anti-NeuN antibody) are shown. (*H*) pS129-α-Syn bundles (*green*) are stained by the autophagy marker p62 (*red*) in neuron cell bodies not in the processes (*arrows*). (*I* and *J*) Concentration-dependent seeding of endogenous α-Syn aggregation is shown. Hippocampal organotypic slices were exposed on day 0 to fibrillar α-Syn-ATTO-550 polymorphs fibrils, ribbons, and fibrils-91 (0.75 or 1.5 μM) and immunolabeled with the anti-pS129-α-Syn antibody 81A on day 14 (*F*). Quantification of percentage area occupied by pS129-α-Syn (*G*) is shown. The box plot shows median, interquartile range, and 10–90% distribution. A Mann-Whitney test was performed to compare the difference between 0.75 and 1.5 μM for each polymorph; the number of images acquired from four experiments (*left* to *right*) was 16, 27, 22, 35, 25, and 31. $^*p < 0.05$, $^{***}p < 0.001$. Scale bars, 10 μM.

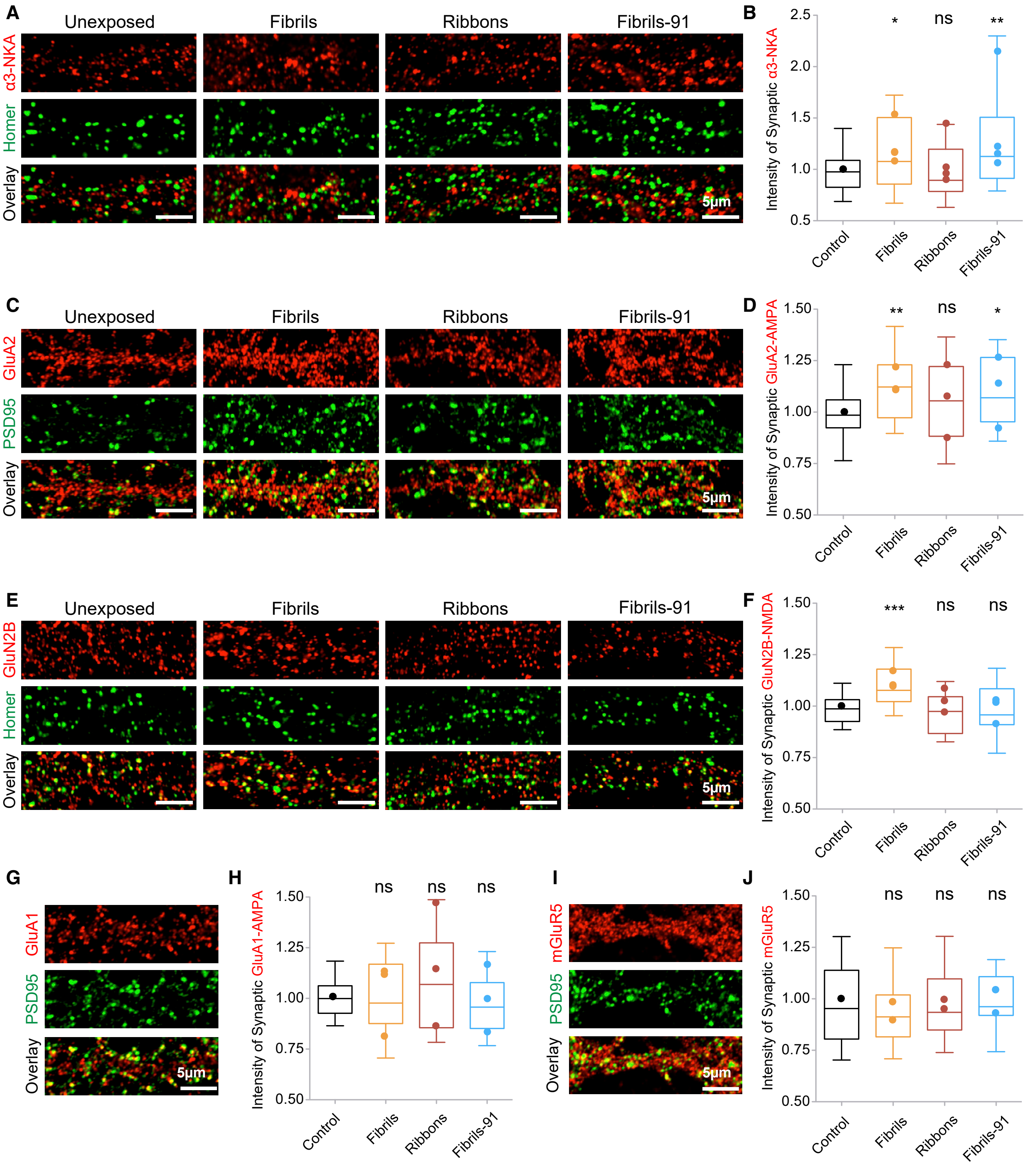

FIGURE 8 Differential redistribution of synaptic α3-NKA, GluA2-AMPA, and GluN2B-NMDA in primary neurons exposed to distinct α-Syn fibrillar polymorphs. (*A–J*) Immunocytochemistry on DIV 21 of (*A*) α3-NKA (*red*) and Homer (*green*), (*C*) GluA2-AMPA receptors (*red*) and PSD95 (*green*), (*E*) GluN2B-NMDA receptors (*red*) and Homer (*green*) (*G*), GluA1-AMPA receptors (*red*) and PSD95 (*green*), and (*I*) mGluR5 receptors (*red*) and Homer (*green*). Primary neurons were exposed (15 min, 250 nM) to the fibrillar α-Syn polymorphs fibrils, ribbons, and fibrils-91 on DIV 14. Quantification of intensity of synaptic α3-NKA (*B*), GluA2-AMPA receptors (*D*), GluN2B-NMDA receptors (*F*), GluA1-AMPA receptors (*H*), and mGluR5 (*J*) clusters (indicative of size; see Material and Methods) was performed and plotted. The box plot shows median, interquartile range, and 10–90% distribution. Number of images (n) analyzed and plotted: (*B*) 47 from four independent experiments, (*D*) 37 from three independent experiments, (*F*) 30 from three independent experiments, (*H*) 37 from three independent experiments, and (*J*) 20 from two independent experiments. A Mann-Whitney test was performed. *$p < 0.05$, **$p < 0.01$, ***$p < 0.001$; ns, not significant. Dot plot shows the averaged value per experiment. Scale bars, 5 μM.

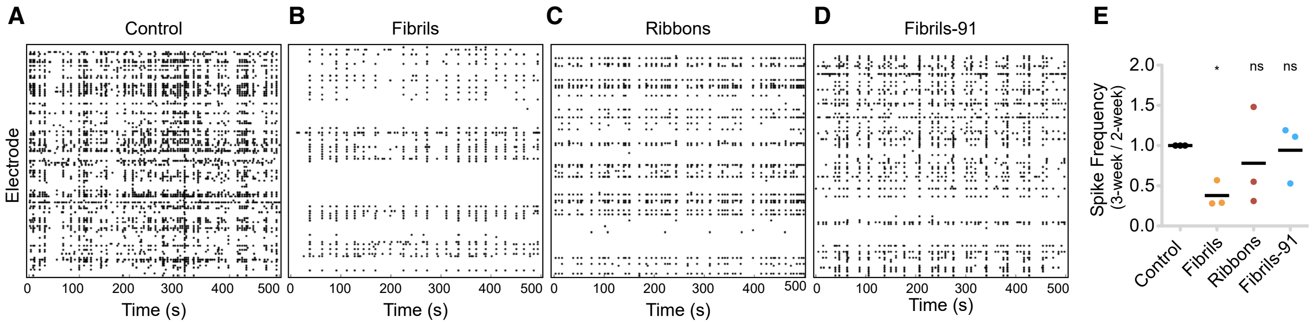

FIGURE 9 Alteration in network activity in primary neurons seeded with α-Syn fibrillar polymorphs. (*A–D*) Raster plots showing the spiking activity of primary neurons (*A*) unexposed or exposed to α-Syn (*B*) fibrils, (*C*) ribbons or (*D*) fibrils 91, recorded using 120-electrode MEA plates seeded with fibrillar α-Syn polymorphs are given. Each row represents one electrode, and each dot represents a single spike obtained on DIV 21 (seeded neurons, 1 week after exposure to α-Syn polymorphs). Notably, control neurons exhibit high spike frequency, whereas fibrillar α-Syn-polymorph-exposed neurons have decreased spiking frequency. (*E*) Quantification showing the normalized ratio of change in spike frequency rate between DIV 14/DIV 21 is given. A nearly 60% reduction in the spike frequency rate is observed in polymorph-fibrils-seeded neurons. Dot plot represents averaged value per experiment. A two-tailed *t*-test to compare difference from control condition was performed. *$p = 0.0227$; ns, not significant; three experiments.

This is consistent with the hypothesis made by Braak for the spread of a pathogen within the central nervous system via neuroanatomical connections in PD (40). We also established that exogenous fibrillar assemblies made of WT α-Syn with distinct structural properties trigger pathological phenotypes characteristic of PD and MSA and imprint their structural characteristics to the monomeric α-Syn they recruit (4,5). This suggests a structure-pathology relationship. We demonstrate here that distinct WT α-Syn fibrillar polymorphs bind to neurons with different efficiencies. We show that the binding, uptake, and seeding of distinct exogenous α-Syn fibrillar polymorphs alter synaptic NMDA, AMPA, and α3-NKA distribution to different extents. We bring evidence for a tight relationship between binding and seeding efficiencies in in vitro and an ex vivo model of synucleinopathy. Indeed, we show that distinct exogenous α-Syn fibrillar polymorphs trigger the aggregation of endogenous monomeric α-Syn to different extents. This indicates that the binding of exogenous fibrillar α-Syn polymorphs to neurons is key for uptake and amplification by seeding. Our findings further establish the function of the plasma membrane as a chemical reactor favoring molecular interactions and the formation of protein clusters by restricting α-Syn fibrillar polymorphs diffusion space from three dimensions to two dimensions (29).

Differential fibrillar α-Syn polymorph binding and clustering in primary and organotypic slice cultures

Intracerebral injection of exogenous α-Syn assemblies is widely used to document their prion-like propagation and the accumulation of pS129-α-Syn (4,6,36,37,41). This experimental approach is nonetheless unsuitable to assess cellular mechanistic processes occurring within short timeframes (hours to days) after the interaction of exogenous α-Syn assemblies with a naïve neuron. Indeed, detectable levels of pathological pS129-α-Syn deposits appear only 2–3 months postinjection, after spine loss begins (42). Primary neuronal culture models represent an alternative to in vivo studies (13). However, most existing protocols are based on the continuous exposure of 5- to 7-day-old neurons that lack mature synapses (43) to exogenous α-Syn assemblies. We therefore developed a robust model based on the use of 14- to 21-day-old primary neurons and backed it up with 14- to 28-day-old organotypic slice cultures, in which neuronal circuitry is maintained, to assess over minutes to 2 weeks the binding and seeding propensities of exogenous fibrillar α-Syn polymorphs and the consequences of these events. We chose hippocampal neurons despite the fact that the striatum and substantia nigra are affected the most in vivo by Lewy pathology. This choice is justified by the fact that seeded aggregation of α-Syn in hippocampal neurons has been well documented (13) and our recent finding that seeding in hippocampal neurons is more efficient than in cortical and striatal counterparts (J. Courte, L. Bousset, Y. von Boxberg, C. Villard, R.M., J.M. Peyrin, unpublished data).

The distinct intrinsic structures fibrillar α-Syn polymorphs exhibit are due to the different conformations monomeric α-Syn adopts within the fibrillar particles. This reflects in their shapes, morphology, proteolytic patterns, and physical properties (5,8,18) and is expected to define their interactomes because distinct polymorphs must expose different amino acid stretches at their surfaces. Recent studies reported the interaction of exogenous fibrillar α-Syn with extracellularly exposed membrane proteins (10,11). The recent cryo-electron microscopy structure for the polymorph fibrils indeed reveals what amino acid stretches and side chains are exposed to the solvent (44). The amino acid stretches that define the interactome of the polymorph fibrils we use here differ from those reported to be exposed to the solvent in fibrillar polymorphs generated by others (45–48). Despite the fact that the amino acid stretches of the distinct fibrillar polymorphs we use

here, with one exception, are unknown, we know they differ because the fibrillar polymorphs amyloid core are unlike (5,8). We demonstrate here that α-Syn polymorph surfaces indeed define their functional properties. They bind differentially to neuronal plasma membranes, cluster, and alter synaptic α3-NKA, NMDA, and AMPA receptor distribution to different extents, minutes to hours after binding and 7 days later when uptake and seeding of endogenous α-Syn has occurred.

Differential fibrillar α-Syn polymorph seeding

Strain-dependent differential seeding is known for various amyloidogenic proteins such as scrapie prions, α-Syn, τ, and amyloid-β (4,6,49,50). We demonstrate here a relationship between binding and seeding. The polymorph that binds best, fibrils-91, seeds to the highest extent. Thus, the differential binding of distinct fibrillar α-Syn polymorphs appears key for their uptake, escape from the endolysosomal compartments (30), and seeding within the cytosol of recipient neurons. Because the distinct fibrillar α-Syn polymorphs were fragmented to have an average length of 40–50 nm (compatible with endocytosis), differential uptake cannot account for the different seeding propensities we report. Because the different polymorphs have different structures, as demonstrated by solid-state NMR measurements (5,8), they expose different amino acid stretches on their surfaces (9). Once taken up, distinct fibrillar polymorphs grow at very different rates because their different ends recruit monomeric α-Syn at rates highly dependent on the abundance of the conformation that can establish highly complementary interactions with their ends. Furthermore, because distinct α-Syn polymorphs expose different amino acid stretches on their sides, they resist clearance to different extents and interact differentially with partner molecules ranging from proteins to lipids. Taken together, the differential binding, growth rates, and resistance of distinct fibrillar α-Syn polymorphs account for their differential accumulation within neurons.

Interestingly, super-resolution imaging revealed that the endogenous, seeded α-Syn aggregates have elongated structures (30–50 nm in width) that bundle over time into structures resembling Lewy neurites. Whether these structures are polymorph specific and possess defined structures remains unclear, and cryo-electron microscopy may allow determining whether, in a manner similar to PolyQ inclusions, they consist of fibrils interacting with cellular endomembranes originating from the endoplasmic reticulum (51).

Functional consequences of the differential interaction of fibrillar α-Syn polymorphs with neuronal membrane components

Synapses are dynamic, and they remodel in an activity- and signaling-dependent manner (52). Differential redistribution of synaptic membrane proteins, but not scaffolds (Homer, PSD), was observed in α-Syn-polymorph-seeded neurons. This suggest that the different polymorphs trigger different molecular signaling pathways. Increased synaptic clustering of α3-NKA was observed upon exposure of neurons to the polymorphs fibrils and fibrils-91, but not ribbons. The redistribution of this pump is deleterious because it prevents the extrusion of sodium ions out of neurons (10,19). Therefore, it is likely that the polymorphs fibrils and fibrils-91 impact a neuron's capacity to extrude sodium ions by forming aberrant clusters. Such aberrant clustering of α3-NKA creates regions within the plasma membrane with reduced local densities of α3-NKA (53).

We previously showed that the α-Syn polymorph fibrils do not interact directly with AMPA and NMDA receptors (10). Nonetheless, this polymorph triggered increased synaptic accumulation of GluA2-subunit containing AMPA and GluN2B-subunit containing NMDA receptors. Thus, the redistribution of AMPA and NMDA is most likely due to homeostatic dysregulation after the perturbation of several signaling pathways (52). The polymorph fibrils-91 also triggered an increase in synaptic accumulation of GluA2-AMPA, but not GluN2B-NMDA, receptors. Fibrils- and fibrils-91-mediated glutamate receptor redistribution at synapses should trigger an enhanced activity-evoked calcium influx and possibly synaptic impairment. No redistribution of those major synaptic components was observed upon exposure of neurons to the polymorph ribbons. This suggests that the fibrillar polymorph ribbons either affects a yet unknown pathway or target other neuronal cells (4). Several pathogenic assemblies—α-Syn (54), amyloid-β (21,22,55), and scrapie prions (14)—have been shown to interact with mGluR5 via PrP^c. None of the fibrillar α-Syn polymorphs we used altered mGluR5 distribution.

Neurons where α-Syn aggregation was seeded by fibrils, but not ribbons and fibrils-91, exhibited gross alteration in neuronal network activity without measurable alterations in synapse density. Neurons exposed to the polymorph fibrils exhibit the lowest pS129-α-Syn load and the strongest redistribution of synaptic receptor. Altogether, our findings suggest that neuronal network activity is most affected by the redistribution of synaptic receptors without measurable alterations in synapse density upon exposure of naïve neurons to pathogenic α-Syn assemblies. This unexpected finding may indicate that synaptic dysfunction and network imbalance precede the appearance of pathology. Contrarily, neurons where α-Syn aggregation was seeded with fibrils-91 displayed extensive pathology but no alteration in network activity. The latter neurons likely compensate for the loss of activity over time but fail to prevent cytosolic aggregation. These aggregates are potential source of "traffic jams" within the cell in which different cytosolic proteins and organelles get trapped.

Altogether, our results suggest a sequential deleterious scenario in which the binding of distinct α-Syn fibrillar

polymorphs to neuron plasma leads to differential redistribution of essential membrane proteins, synaptic remodeling, and impaired neuronal activity. After uptake, distinct α-Syn fibrillar polymorphs further trigger noxious changes with the differential seeded aggregation of endogenous α-Syn and the impact this has on normal cytosolic trafficking and mitochondrial function (24,56,57). This is of particular interest in a context in which recent reports bring solid evidence for the existence of α-Syn polymorphs in the brains of patients who developed distinct synucleinopathies (58) and for their capacity to selectively target brain regions and cells types from the central nervous system (59,60). Our results, together with recent reports using patient-derived pathogenic α-Syn, highlight the importance of targeting α-Syn aggregates' prion-like propagation in therapeutic approaches to synucleinopathies. As for de novo-generated α-Syn fibrillar polymorphs, the future assessment of patient-derived pathogenic α-Syn aggregates' ability, amplified ex vivo or not, to differentially redistribute neuron membrane proteins, remodel synapses, and seed the aggregation of α-Syn in neurons may bring novel insights into synucleinopathies.

Overall, we demonstrate here that α-Syn polymorphs' surfaces define their binding and seeding propensity, with subsequent differential redistribution of partner proteins at synapses and consequences for neuronal network activity. These findings are consistent with the view that distinct synucleinopathies may result from the changes in neuronal membrane and cytosolic protein homeostasis different α-Syn polymorphs trigger.

SUPPORTING MATERIAL

Supporting Material can be found online at https://doi.org/10.1016/j.bpj.2020.01.022.

AUTHOR CONTRIBUTIONS

Conceived the project and designed experiment: A.N.S. and R.M.; performed experiments and analyzed data: A.N.S., L.B., M.R., J.S., and V.R.; provided resources, funding, and equipment: R.M. and A.T.; wrote the manuscript: A.N.S., A.T., and R.M.

ACKNOWLEDGMENTS

The authors thank Tracy Bellande and Margaux Petay for expert technical assistance. We thank Luke D. Lavis for providing Janelia Farm dyes and Maria J. Pinto for assistance in slice culture work.

This work was supported by grants from the EC Joint Programme on Neurodegenerative Diseases (TransPathND, ANR-17-JPCD-0002-02); the Centre National de la Recherche Scientifique; The Fondation pour la Recherche Médicale (contract DEQ 20160334896); a "Coup d'Elan a la Recherche Francaise" award from Fondation Bettencourt-Schueller; the Fondation Simone et Cino Del Duca of the Institut de France; the European Union's Horizon 2020 Research and Innovation Programme and EFPIA Innovative Medicines Initiative 2 under grant agreements nos. 116060 (IMPRiND) and 821522 (PD-MitoQUANT); an ERC advanced research grant (PLASLTINHIB); and the "Investissements d'Avenir" program (ANR-10-LABX-54 MEMO LIFE and ANR-11-IDEX-0001-02, PSL Research University). This work benefited from the JiePie research award attributed to R.M., the electron microscopy facility Imagerie-Gif, and the proteomic facility SICaPS. A.N.S. is currently an employee at U.C.B. Biopharma SRL.

REFERENCES

1. Wong, Y. C., and D. Krainc. 2017. α-synuclein toxicity in neurodegeneration: mechanism and therapeutic strategies. *Nat. Med.* 23:1–13.
2. Melki, R. 2015. Role of different alpha-synuclein strains in synucleinopathies, similarities with other neurodegenerative diseases. *J. Parkinsons Dis.* 5:217–227.
3. Melki, R. 2017. The multitude of therapeutic targets in neurodegenerative proteinopathies. *In* Disease-Modifying Targets in Neurodegenerative Disorders. V. Baekelandt and E. Lobbestael, eds. Elsevier, pp. 1–20.
4. Peelaerts, W., L. Bousset, ..., V. Baekelandt. 2015. α-Synuclein strains cause distinct synucleinopathies after local and systemic administration. *Nature.* 522:340–344.
5. Bousset, L., L. Pieri, ..., R. Melki. 2013. Structural and functional characterization of two alpha-synuclein strains. *Nat. Commun.* 4:2575.
6. Guo, J. L., D. J. Covell, ..., V. M. Y. Lee. 2013. Distinct α-synuclein strains differentially promote tau inclusions in neurons. *Cell.* 154:103–117.
7. Prusiner, S. B., A. L. Woerman, ..., K. Giles. 2015. Evidence for α-synuclein prions causing multiple system atrophy in humans with parkinsonism. *Proc. Natl. Acad. Sci. USA.* 112:E5308–E5317.
8. Verasdonck, J., L. Bousset, ..., B. H. Meier. 2016. Further exploration of the conformational space of α-synuclein fibrils: solid-state NMR assignment of a high-pH polymorph. *Biomol. NMR Assign.* 10:5–12.
9. Melki, R. 2018. How the shapes of seeds can influence pathology. *Neurobiol. Dis.* 109:201–208.
10. Shrivastava, A. N., V. Redeker, ..., A. Triller. 2015. α-synuclein assemblies sequester neuronal α3-Na+/K+-ATPase and impair Na+ gradient. *EMBO J.* 34:2408–2423.
11. Holmes, B. B., S. L. DeVos, ..., M. I. Diamond. 2013. Heparan sulfate proteoglycans mediate internalization and propagation of specific proteopathic seeds. *Proc. Natl. Acad. Sci. USA.* 110:E3138–E3147.
12. Brundin, P., R. Melki, and R. Kopito. 2010. Prion-like transmission of protein aggregates in neurodegenerative diseases. *Nat. Rev. Mol. Cell Biol.* 11:301–307.
13. Volpicelli-Daley, L. A., K. C. Luk, ..., V. M.-Y. Lee. 2011. Exogenous α-synuclein fibrils induce Lewy body pathology leading to synaptic dysfunction and neuron death. *Neuron.* 72:57–71.
14. Goniotaki, D., A. K. K. Lakkaraju, ..., A. Aguzzi. 2017. Inhibition of group-I metabotropic glutamate receptors protects against prion toxicity. *PLoS Pathog.* 13:e1006733.
15. Falsig, J., T. Sonati, ..., A. Aguzzi. 2012. Prion pathogenesis is faithfully reproduced in cerebellar organotypic slice cultures. *PLoS Pathog.* 8:e1002985.
16. Sonati, T., R. R. Reimann, ..., A. Aguzzi. 2013. The toxicity of antiprion antibodies is mediated by the flexible tail of the prion protein. *Nature.* 501:102–106.
17. Ghee, M., R. Melki, ..., J. Mallet. 2005. PA700, the regulatory complex of the 26S proteasome, interferes with alpha-synuclein assembly. *FEBS J.* 272:4023–4033.
18. Makky, A., L. Bousset, ..., R. Melki. 2016. Nanomechanical properties of distinct fibrillar polymorphs of the protein α-synuclein. *Sci. Rep.* 6:37970.

19. Shrivastava, A. N., V. Redeker, …, R. Melki. 2019. Clustering of Tau fibrils impairs the synaptic composition of α3-Na^+/K^+-ATPase and AMPA receptors. *EMBO J.* 38:e99871.

20. Cantaut-Belarif, Y., M. Antri, …, A. Bessis. 2017. Microglia control the glycinergic but not the GABAergic synapses via prostaglandin E2 in the spinal cord. *J. Cell Biol.* 216:2979–2989.

21. Renner, M., P. N. Lacor, …, A. Triller. 2010. Deleterious effects of amyloid β oligomers acting as an extracellular scaffold for mGluR5. *Neuron.* 66:739–754.

22. Shrivastava, A. N., J. M. Kowalewski, …, A. Triller. 2013. β-amyloid and ATP-induced diffusional trapping of astrocyte and neuronal metabotropic glutamate type-5 receptors. *Glia.* 61:1673–1686.

23. Bannai, H., F. Niwa, …, K. Mikoshiba. 2015. Bidirectional control of synaptic GABAAR clustering by glutamate and calcium. *Cell Rep.* 13:2768–2780.

24. Gribaudo, S., P. Tixador, …, A. L. Perrier. 2019. Propagation of α-synuclein strains within human reconstructed neuronal network. *Stem Cell Rep.* 12:230–244.

25. Malkusch, S., and M. Heilemann. 2016. Extracting quantitative information from single-molecule super-resolution imaging data with LAMA - LocAlization Microscopy Analyzer. *Sci. Rep.* 6:34486.

26. Ester, M., H.-P. Kriegel, …, X. Xu. 1996. A density-based algorithm for discovering clusters in large spatial databases with noise. *In* KDD'96 Proceedings of the Second International Conference on Knowledge Discovery and Data Mining. E. Simoudis, J. Han, and U. Fayyad, eds. AAI Press, pp. 226–231.

27. Grimm, J. B., B. P. English, …, L. D. Lavis. 2016. Bright photoactivatable fluorophores for single-molecule imaging. *Nat. Methods.* 13:985–988.

28. Persson, F., M. Lindén, …, J. Elf. 2013. Extracting intracellular diffusive states and transition rates from single-molecule tracking data. *Nat. Methods.* 10:265–269.

29. Shrivastava, A. N., A. Aperia, …, A. Triller. 2017. Physico-pathologic mechanisms involved in neurodegeneration: misfolded protein-plasma membrane interactions. *Neuron.* 95:33–50.

30. Flavin, W. P., L. Bousset, …, E. M. Campbell. 2017. Endocytic vesicle rupture is a conserved mechanism of cellular invasion by amyloid proteins. *Acta Neuropathol.* 134:629–653.

31. Abounit, S., L. Bousset, …, C. Zurzolo. 2016. Tunneling nanotubes spread fibrillar α-synuclein by intercellular trafficking of lysosomes. *EMBO J.* 35:2120–2138.

32. Ivenshitz, M., and M. Segal. 2010. Neuronal density determines network connectivity and spontaneous activity in cultured hippocampus. *J. Neurophysiol.* 104:1052–1060.

33. Unni, V. K., T. A. Weissman, …, B. T. Hyman. 2010. In vivo imaging of alpha-synuclein in mouse cortex demonstrates stable expression and differential subcellular compartment mobility. *PLoS One.* 5:e10589.

34. Elmore, M. R. P., A. R. Najafi, …, K. N. Green. 2014. Colony-stimulating factor 1 receptor signaling is necessary for microglia viability, unmasking a microglia progenitor cell in the adult brain. *Neuron.* 82:380–397.

35. Rey, N. L., J. A. Steiner, …, P. Brundin. 2016. Widespread transneuronal propagation of α-synucleinopathy triggered in olfactory bulb mimics prodromal Parkinson's disease. *J. Exp. Med.* 213:1759–1778.

36. Hansen, C., E. Angot, …, P. Brundin. 2011. α-Synuclein propagates from mouse brain to grafted dopaminergic neurons and seeds aggregation in cultured human cells. *J. Clin. Invest.* 121:715–725.

37. Luk, K. C., V. Kehm, …, V. M.-Y. Lee. 2012. Pathological α-synuclein transmission initiates Parkinson-like neurodegeneration in nontransgenic mice. *Science.* 338:949–953.

38. Freundt, E. C., N. Maynard, …, M. Brahic. 2012. Neuron-to-neuron transmission of α-synuclein fibrils through axonal transport. *Ann. Neurol.* 72:517–524.

39. Brahic, M., L. Bousset, …, A. D. Gitler. 2016. Axonal transport and secretion of fibrillar forms of α-synuclein, Aβ42 peptide and HTTExon 1. *Acta Neuropathol.* 131:539–548.

40. Braak, H., K. Del Tredici, …, E. Braak. 2003. Staging of brain pathology related to sporadic Parkinson's disease. *Neurobiol. Aging.* 24:197–211.

41. Mougenot, A.-L., S. Nicot, …, T. Baron. 2012. Prion-like acceleration of a synucleinopathy in a transgenic mouse model. *Neurobiol. Aging.* 33:2225–2228.

42. Blumenstock, S., E. F. Rodrigues, …, J. Herms. 2017. Seeding and transgenic overexpression of alpha-synuclein triggers dendritic spine pathology in the neocortex. *EMBO Mol. Med.* 9:716–731.

43. Ichikawa, M., K. Muramoto, …, Y. Kuroda. 1993. Formation and maturation of synapses in primary cultures of rat cerebral cortical cells: an electron microscopic study. *Neurosci. Res.* 16:95–103.

44. Guerrero-Ferreira, R., N. M. Taylor, …, H. Stahlberg. 2019. Two new polymorphic structures of human full-length alpha-synuclein fibrils solved by cryo-electron microscopy. *eLife.* 8:e48907.

45. Li, B., P. Ge, …, L. Jiang. 2018. Cryo-EM of full-length α-synuclein reveals fibril polymorphs with a common structural kernel. *Nat. Commun.* 9:3609.

46. Rodriguez, J. A., M. I. Ivanova, …, D. S. Eisenberg. 2015. Structure of the toxic core of α-synuclein from invisible crystals. *Nature.* 525:486–490.

47. Tuttle, M. D., G. Comellas, …, C. M. Rienstra. 2016. Solid-state NMR structure of a pathogenic fibril of full-length human α-synuclein. *Nat. Struct. Mol. Biol.* 23:409–415.

48. Li, Y., C. Zhao, …, X. Li. 2018. Amyloid fibril structure of α-synuclein determined by cryo-electron microscopy. *Cell Res.* 28:897–903.

49. Aguzzi, A., M. Heikenwalder, and M. Polymenidou. 2007. Insights into prion strains and neurotoxicity. *Nat. Rev. Mol. Cell Biol.* 8:552–561.

50. Kaufman, S. K., D. W. Sanders, …, M. I. Diamond. 2016. Tau prion strains dictate patterns of cell pathology, progression rate, and regional vulnerability in vivo. *Neuron.* 92:796–812.

51. Bäuerlein, F. J. B., I. Saha, …, R. Fernández-Busnadiego. 2017. In situ architecture and cellular interactions of PolyQ inclusions. *Cell.* 171:179–187.e10.

52. Choquet, D., and A. Triller. 2013. The dynamic synapse. *Neuron.* 80:691–703.

53. Shrivastava, A. N., A. Triller, and R. Melki. 2018. Cell biology and dynamics of Neuronal Na^+/K^+-ATPase in health and diseases. *Neuropharmacology.* 13:107461.

54. Ferreira, D. G., M. Temido-Ferreira, …, T. F. Outeiro. 2017. α-synuclein interacts with PrP^C to induce cognitive impairment through mGluR5 and NMDAR2B. *Nat. Neurosci.* 20:1569–1579.

55. Um, J. W., A. C. Kaufman, …, S. M. Strittmatter. 2013. Metabotropic glutamate receptor 5 is a coreceptor for Alzheimer aβ oligomer bound to cellular prion protein. *Neuron.* 79:887–902.

56. Valdinocci, D., R. F. Simões, …, D. L. Pountney. 2019. Intracellular and intercellular mitochondrial dynamics in Parkinson's disease. *Front. Neurosci.* 13:930.

57. Ordonez, D. G., M. K. Lee, and M. B. Feany. 2018. α-synuclein induces mitochondrial dysfunction through spectrin and the actin cytoskeleton. *Neuron.* 97:108–124.e6.

58. Strohäker, T., B. C. Jung, …, M. Zweckstetter. 2019. Structural heterogeneity of α-synuclein fibrils amplified from patient brain extracts. *Nat. Commun.* 10:5535.

59. Lau, A., R. W. L. So, …, J. C. Watts. 2020. α-Synuclein strains target distinct brain regions and cell types. *Nat. Neurosci.* 23:21–31.

60. Rey, N. L., L. Bousset, …, P. Brundin. 2019. α-Synuclein conformational strains spread, seed and target neuronal cells differentially after injection into the olfactory bulb. *Acta Neuropathol. Commun.* 7:221.

Biophysical Journal, Volume *118*

Supplemental Information

Differential Membrane Binding and Seeding of Distinct α-Synuclein Fibrillar Polymorphs

Amulya Nidhi Shrivastava, Luc Bousset, Marianne Renner, Virginie Redeker, Jimmy Savistchenko, Antoine Triller, and Ronald Melki

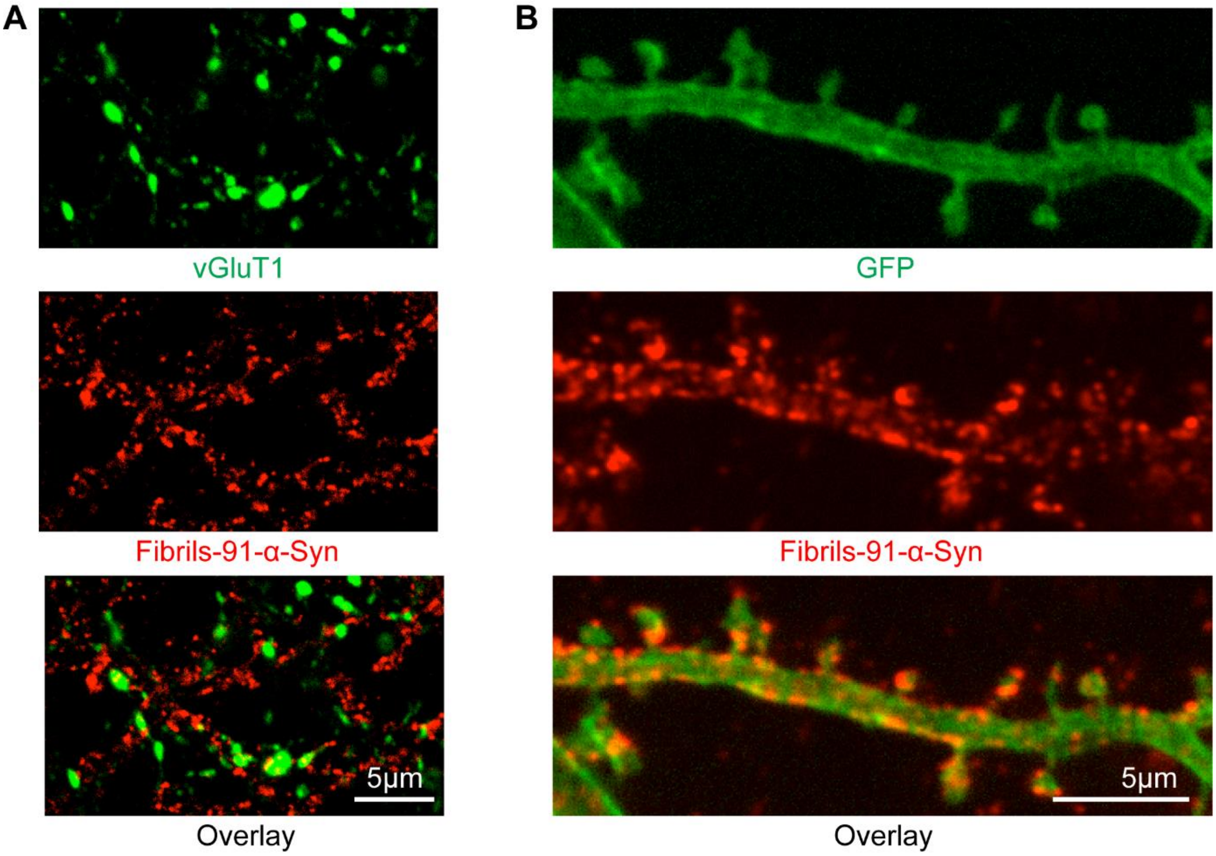

Supplementary Figure 1. Binding of α-Syn in and out of synapses

(**A**) Exposure of neurons to fibrillar α-Syn polymorphs (image for Fibrils-91-ATTO550 is presented, red) followed by immunolabeling of excitatory pre-synaptic boutons using vGluT1 (green) antibody. Binding and clustering of α-Syn is observed both in and out of synaptic terminals.

(**B**) Binding of α-Syn polymorphs (image for Fibrils-91-ATTO550 is presented, red) on neuronal dendrite (labeled with GFP, green). Binding and clustering of α-Syn is observed both in dendritic spines and shaft.

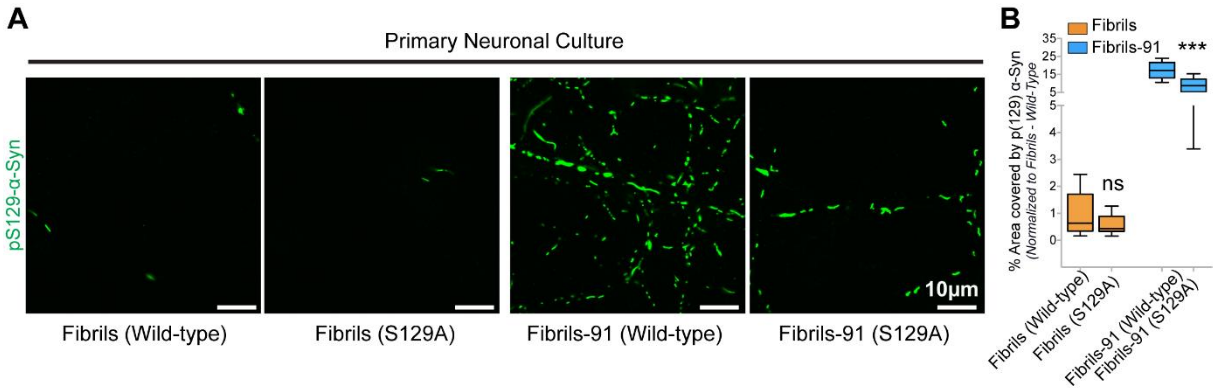

Supplementary Figure 2. Endogenous α-Syn is phosphorylated upon exposure in primary neuronal cultures to exogenous fibrillar polymorphs

(**A-B**) Primary mature hippocampal neurons cultures were exposed to wild-type or S129A fibrillar polymorphs (Fibrils and Fibrils-91) (250nM, 15 min in fresh culture medium) at DIV 14. After extensive washing, cells were transferred back to the original culture medium. Neurons were fixed at DIV 21 and immunolabeled for pS129-α-Syn (**A**). Similar pS129-α-Syn signal was observed after exposure to wild-type or S129A fibrillar α-Syn polymorphs (**A**). Quantification of the area occupied by aggregated pS129-α-Syn using the 81A antibody following exposure to the fibrillar α-Syn polymorphs.

Box-plot shows median, inter-quartile range and 10-90% distribution. Mann-Whitney test is performed to compare the difference; number of images (n) from 2 experiments (left to right): 16, 22, 30, 30. ***p<0.001, ns= not significant.

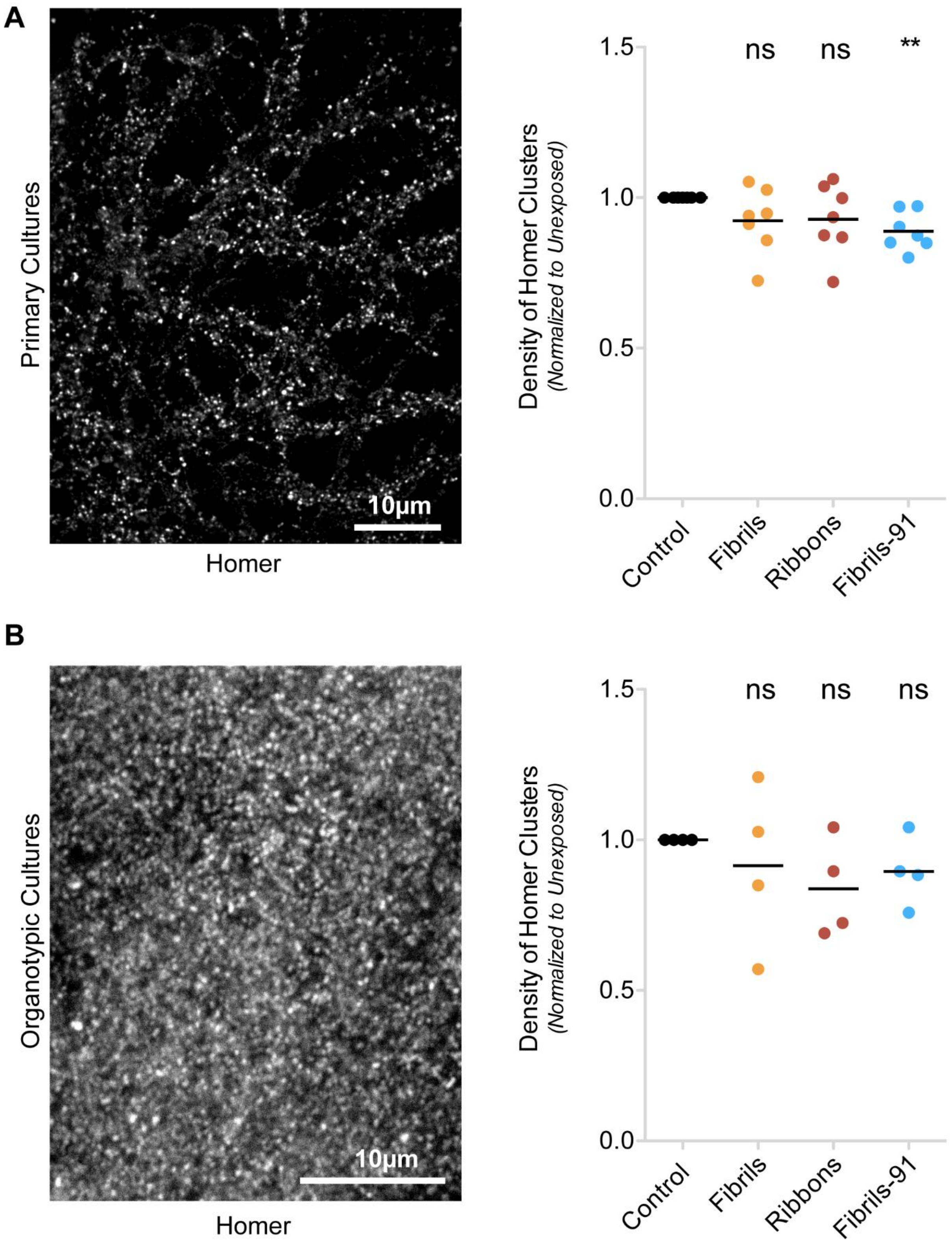

Supplementary Figure 3. No alteration in synapse density in α-Syn seeded neurons

(**A-D**) Immunodetection of synapses using Homer antibody in primary neurons (**A**) or organotypic slices (**B**) in control or α-Syn polymorphs seeded neurons. No or subtle alteration in synaptic density was observed (**B, D**). Dot plot shows average value from 7-experiments in cultures and 4-experiments in slices; Mann-Whitney test, **p<0.01, ns= not significant.

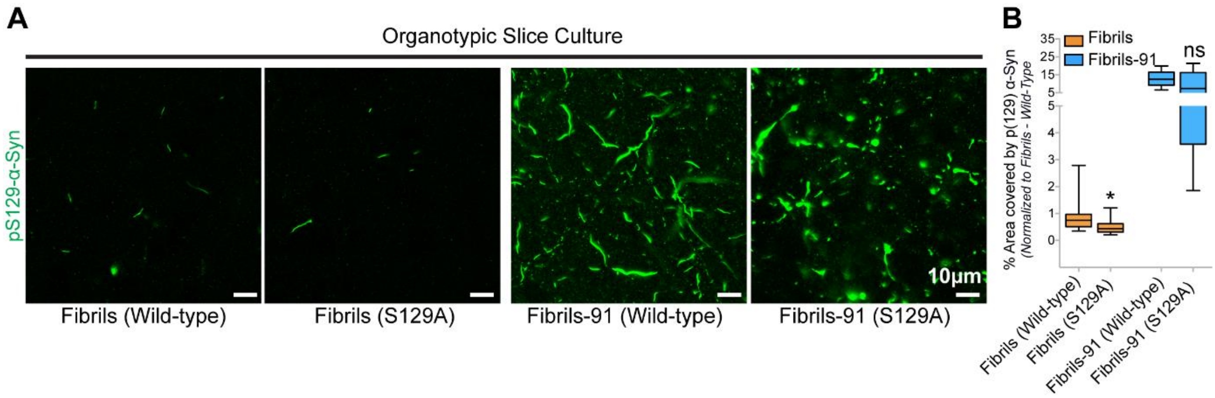

Supplementary Figure 4. Endogenous α-Syn is phosphorylated upon exposure of organotypic slice cultures to exogenous fibrillar polymorphs

(**A-B**) Organotypic slice cultures were exposed to wild-type or S129A fibrillar polymorphs (Fibrils and Fibrils-91 polymorphs) (1.5µM, 15 min in fresh culture medium) at day 0. After extensive washing, slices were transferred to a new culture medium. Slices were fixed on day 14 and immunolabeled for pS129-α-Syn (**A**). Similar pS129-α-Syn signal was observed after exposure to wild-type or S129A fibrillar α-Syn polymorphs (**A**). Quantification of the area occupied by aggregated pS129-α-Syn using the 81A antibody following exposure to the fibrillar α-Syn polymorphs.

Box-plot shows median, inter-quartile range and 10-90% distribution. Mann-Whitney test is performed to compare the difference; number of images (n) from 2 experiments (left to right): 22, 21, 22, 23. *$p<0.05$, ns= not significant.

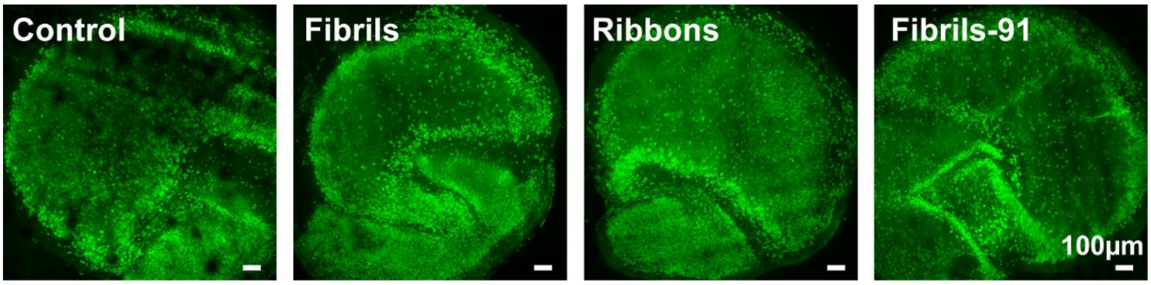

Supplementary Figure 5. Morphology of organotypic slices in seeded neurons

Low magnification images of brain slices immunostained for neuronal marker, NeuN (green) prior (control) or after exposure to α-Syn polymorphs on Day 28 in culture after addition of the fibrillar polymorphs on Day 14. No alteration in hippocampal morphology is detected.

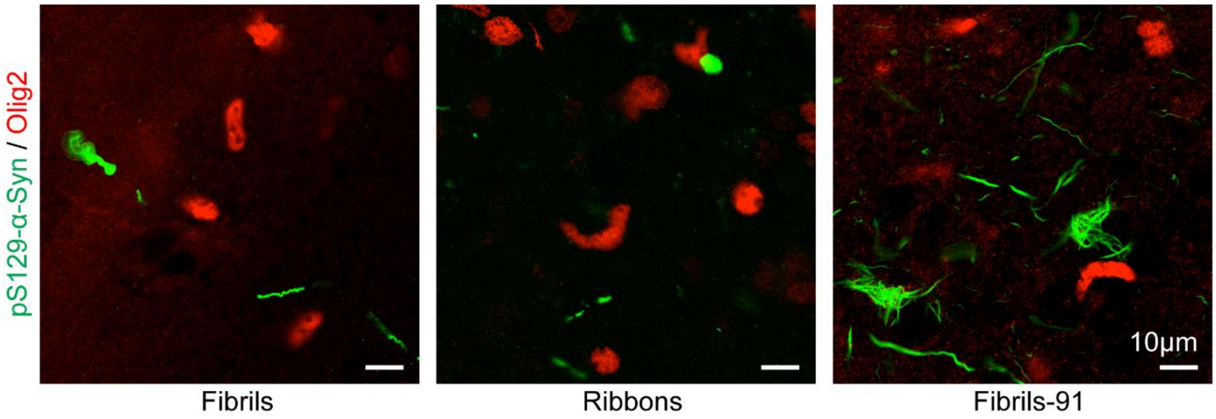

Supplementary Figure 6. Detection of pS129-α-Syn aggregates in oligodendrocytes

Organotypic slice cultures were exposed to the fibrillar polymorphs Fibrils, Ribbons and Fibrils-91(1.5µM) at day 0. Slices were fixed on day 14 and immunolabeled for pS129-α-Syn (81A antibody) and oligodendrocyte marker (Olig 2 antibody). No pS129-α-Syn deposits in Olig2 positive cells for slices exposed to Fibrils/Fibrils-91 polymorphs was observed. Occasional pS129-α-Syn reactivity within Olig2-positive oligodendrocytes was observed for Ribbons (middle).

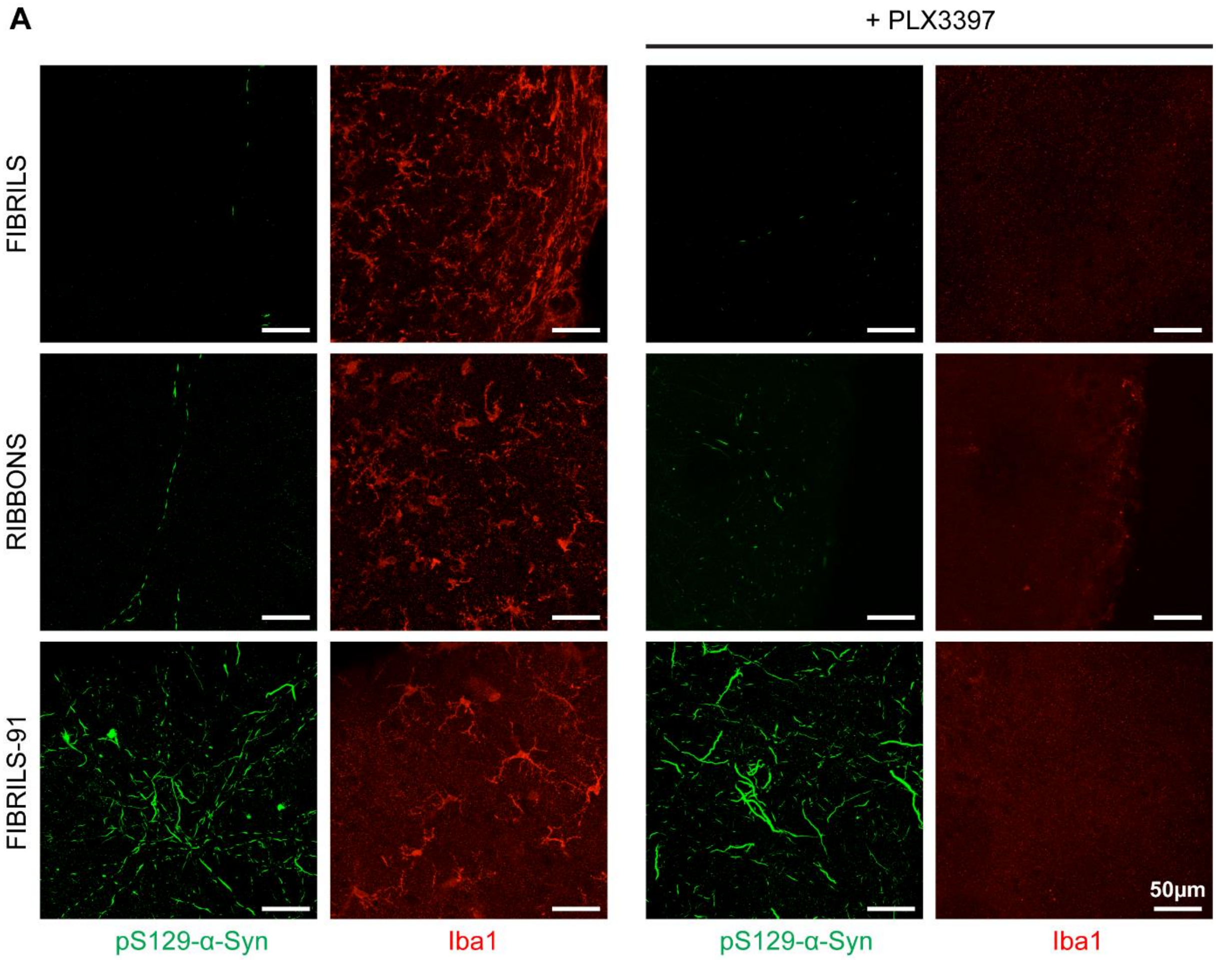
A
+ PLX3397
FIBRILS
RIBBONS
FIBRILS-91
50µm
pS129-α-Syn
Iba1
pS129-α-Syn
Iba1

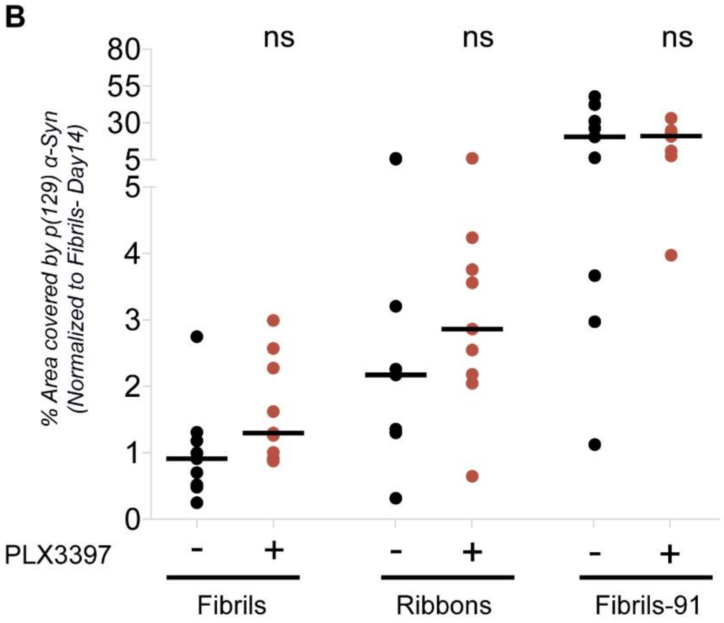
B
ns
ns
ns
% Area covered by p(129) α-Syn
(Normalized to Fibrils- Day14)
80
55
30
5
5
4
3
2
1
0
PLX3397
-
+
-
+
-
+
Fibrils
Ribbons
Fibrils-91

Supplementary Figure 7. Unaltered seeding in organotypic slices following microglia depletion

(**A-B**) Organotypic slice cultures were prepared (day -14) and exposed to α-Syn fibrillar polymorphs (1.5µM, 15 min in fresh culture medium) at day 0. After extensive washing, the slices were transferred to a new culture medium. Slices were fixed on day 14 and immunolabeled for pS129-α-Syn and Iba1. Complete microglia depletion (**A**) was achieved using PLX3397 treatment as described in Materials and Methods. Notably fibrillar polymorphs seeded the aggregation of endogenous α-Syn both in absence and presence of microglia (**A, B**). Dot-plot shows individual organotypic slice. Mann-Whitney test is performed to compare the difference; number of slices (n=10) from 3 experiments. *$p<0.05$, ns= not significant.

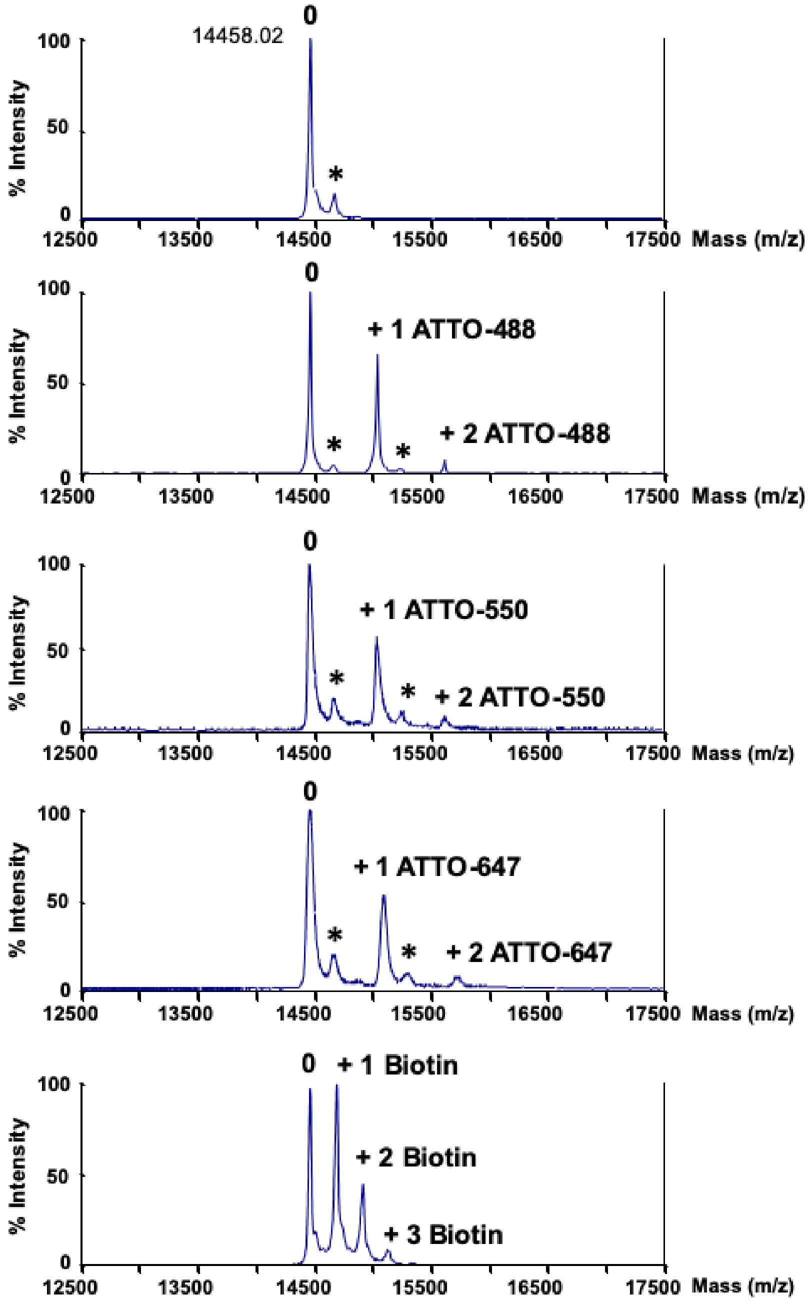
0
14458.02
100
50
0
% Intensity
*
12500
13500
14500
15500
16500
17500
Mass (m/z)
0
+ 1 ATTO-488
+ 2 ATTO-488
0
+ 1 ATTO-550
+ 2 ATTO-550
0
+ 1 ATTO-647
+ 2 ATTO-647
0
+ 1 Biotin
+ 2 Biotin
+ 3 Biotin

Supplementary Figure 8. Quality control analysis of labeled α-Syn Fibrils . MALDI-TOF mass spectra, from top to bottom of (A) unlabelled, (B) ATTO480, (C) ATTO550, (D) ATTO647 and (E) biotin-labeled wild-type α-Syn Fibrils are shown. The spectra show that α-Syn is labelled on average by ≤ one ATTO480, 550, 647 or biotin molecule. Stars depict sinapinic acid matrix adducts.

For mass spectrometry analysis, the samples were de-salted (with 5% acetonitrile, 0.1% Trifluoroacetic acid (TFA)) and eluted from a C18 reversed-phase Zip-Tip (Millipore, Billerica, MA, USA) in 50% acetonitrile, 0.1% TFA. The polypeptides were mixed in a ratio of 1:5 to 1:20 (v⁄v) with sinapinic acid (10 mg/mL) in 50% acetonitrile and 0.1% TFA) and spotted (0.5 µL) on a stainless steel MALDI target (Opti-TOF; Applied BioSystems). MALDI-TOF-TOF MS spectra were acquired with a MALDI-TOF⁄TOF 5800 mass spectrometer (Applied Biosystems) using linear mode acquisition. Acquisition and data analysis were performed using the Data Explorer software from Applied Biosystems.
